# Comparative efficacy of sodium-glucose cotransporter-2 inhibitors (SGLT2i) for cardiovascular outcomes in type 2 diabetes: a systematic review and network meta-analysis of randomised controlled trials

**DOI:** 10.1007/s10741-020-09954-8

**Published:** 2020-04-20

**Authors:** Tobias Täger, Dan Atar, Stefan Agewall, Hugo A. Katus, Morten Grundtvig, John G. F. Cleland, Andrew L. Clark, Hanna Fröhlich, Lutz Frankenstein

**Affiliations:** 1 Department of Cardiology, Angiology, and Pulmonology, University Hospital Heidelberg, Im Neuenheimer Feld 410, 69120 Heidelberg, Germany; 2 Department of Cardiology, Oslo University Hospital, Ulleval and Institute of Clinical Sciences, University of Oslo, Oslo, Norway; 3 Medical Department, Innlandet Hospital Trust Division Lillehammer, Lillehammer, Norway; 4 National Heart & Lung Institute, Royal Brompton & Harefield Hospitals, Imperial College, London, and Robertson Centre for Biostatistics & Clinical Trials, Glasgow, UK; 5 Castle Hill Hospital of the University of Hull, Cottingham, UK

**Keywords:** Sodium-glucose cotransporter-2 inhibitors, Type 2 diabetes, Mortality, Heart failure, Efficacy

## Abstract

Sodium-glucose cotransporter-2 inhibitors (SGLT2i) improve cardiovascular outcomes in patients with type 2 diabetes mellitus (T2D). The comparative efficacy of individual SGLT2i remains unclear. We searched PubMed, www.clinicaltrials.gov and the Cochrane Central Register of Controlled Trials for randomised controlled trials exploring the use of canagliflozin, dapagliflozin, empagliflozin or ertugliflozin in patients with T2D. Comparators included placebo or any other active treatment. The primary endpoint was all-cause mortality. Secondary endpoints were cardiovascular mortality and worsening heart failure (HF). Evidence was synthesised using network meta-analysis (NMA). Sixty-four trials reporting on 74,874 patients were included. The overall quality of evidence was high. When compared with placebo, empagliflozin and canagliflozin improved all three endpoints, whereas dapagliflozin improved worsening HF. When compared with other SGLT2i, empagliflozin was superior for all-cause and cardiovascular mortality reduction. Empagliflozin, canagliflozin and dapagliflozin had similar effects on improving worsening HF. Ertugliflozin had no effect on any of the three endpoints investigated. Sensitivity analyses including extension periods of trials or excluding studies with a treatment duration of < 52 weeks confirmed the main results. Similar results were obtained when restricting mortality analyses to patients included in cardiovascular outcome trials (*n* = 38,719). Empagliflozin and canagliflozin improved survival with empagliflozin being superior to the other SGLT2i. Empagliflozin, canagliflozin and dapagliflozin had similar effects on improving worsening HF. Prospective head-to-head comparisons would be needed to confirm these results.

**Electronic supplementary material:**

The online version of this article (10.1007/s10741-020-09954-8) contains supplementary material, which is available to authorized users.

## Introduction

Sodium-glucose cotransporter-2 inhibitors (SGLT2i) are a new class of oral anti-diabetic drugs (OAD) with a moderate effect on glycaemic control and a low risk of hypoglycaemia and weight gain [1, 2]. Current evidence suggests that SGLT2i improve cardiovascular endpoints including all-cause mortality, cardiovascular mortality, heart failure (HF) and atherosclerotic macrovascular events [3]. The magnitude of cardiovascular risk reduction with SGLT2i, however, differed between trials [3–6]. Furthermore, there is concern regarding the potential cardiovascular safety of some OAD [7]. There is thus remaining uncertainty about the comparative efficacy of individual SGLT2i or whether a class effect can be assumed. To date, there are no prospective or retrospective head-to-head comparisons of individual SGLT2i. Given the required sample size and associated costs, a comparative SGLT2i trial may never be done. We therefore performed a network meta-analysis (NMA) of randomised controlled trials to compare comprehensively the cardiovascular benefits of SGLT2i in patients with type 2 diabetes mellitus (T2D).

## Methods

NMA is an extension of pairwise meta-analysis in which multiple treatments are being compared using both direct comparisons of interventions within randomised controlled trials and indirect comparisons across trials based on a common comparator. NMA has advantages over pairwise meta-analysis, such as clarification of inconsistent outcomes from multiple studies including multiple common comparators and indirect effect calculation of missing direct comparisons between important treatments. Also, NMA can provide increased statistical power and cross-validation of the observed treatment effect of weak connections with reasonable network connectivity and sufficient sample sizes. This results in greater precision of treatment effect estimates and the ability to rank all the interventions in a coherent way.

We performed the present review following the Preferred Reporting Items for Systematic Reviews and Meta-Analyses (PRISMA) extension statement for reporting systematic reviews incorporating NMAs of health care interventions [8–11]. The protocol of the NMA was prospectively registered at final registration ID at PROSPERO: CRD42020151112.

### Identification and selection of studies

We searched electronic databases (PubMed, Cochrane Central Register of Controlled Trials) and websites (www.clinicaltrials.gov) up to August 12, 2019 for randomised controlled trials investigating the use of canagliflozin, dapagliflozin, empagliflozin or ertugliflozin in patients with T2D. Details of the search strategy are provided in the *supplemental material*. In addition, reviews and meta-analyses of SGLT2i published in PubMed between 2017 and 2019 were screened for additional SGLT2i trials. Two reviewers independently screened citations against the following predefined selection criteria.

#### Study design

Prospective randomised controlled trials with either parallel-group (all endpoints) or cross-over design (worsening heart failure (HF) only) were included. There were no restrictions regarding date of publication, language or sample size.

#### Population

We included *s*tudies evaluating adults (≥ 18 years) with a diagnosis of T2D and treatment with SGLT2i for at least 24 weeks. There were no restrictions regarding sex, race, background diabetes treatment or dose of SGLT2i.

#### Interventions

Treatment was with either canagliflozin, dapagliflozin, empagliflozin or ertugliflozin for at least 24 weeks. This arbitrary limit of 24 weeks was chosen to allow a potential survival benefit to become detectable against the overall low short-term baseline mortality in diabetic cohorts. Analyses were restricted to canagliflozin, dapagliflozin, empagliflozin and ertugliflozin since these agents have been approved by both the United States Food and Drug Administration and the European Medicines Agency.

#### Comparators

Placebo or standard medical care.

#### Outcomes

Primary outcome was all-cause mortality. Secondary outcomes included cardiovascular mortality and worsening HF.

### Data extraction and quality assessment

All relevant articles were independently reviewed by two investigators to assess the eligibility of the article and abstract with standardised data abstraction forms, and disagreement was resolved by a third investigator. For each trial included, details were extracted on study design, patient characteristics, interventions and outcomes. The quality of included trials was assessed using the Cochrane Collaboration Criteria [12].

### Statistical analyses

This NMA was conducted with Stata software 15.0 (StataCorp, College Station, TX, USA) using the network family of commands [13, 14]. A random effects model was applied. The NMA was performed to obtain estimates for outcomes of primary and secondary endpoints, presented as relative risks (RR) and 95% confidence intervals (CI) for binary outcomes. The plot of a network of drugs was used as a visual representation of the evidence base and offered a concise description of its characteristics. It consists of nodes representing the drugs being compared and edges representing the available direct comparisons (comparisons evaluated in at least one study) between pairs of drugs [14–16]. The quality of treatment effect estimates was rated following the Grading of Recommendations Assessment, Development and Evaluation (GRADE) approach [17, 18]. In order to make the rank of treatments, we used the surface under the cumulative ranking probabilities (SUCRA)—a transformation of the mean rank that accounts both for the location and the variance of all relative treatment effects [19]. SUCRA values range from 0 to 1.0. The higher the SUCRA value, and the closer to 1.0, the higher the likelihood that a therapy is in the top rank or one of the top ranks; the closer to 0 the SUCRA value, the more likely that a therapy is in the bottom rank, or one of the bottom ranks [20]. To check for a publication bias, we designed a funnel plot [14]. Consistency of results was evaluated in each loop by calculation of an inconsistency factor and statistical significance determined via z-test [16, 21].

For trials comprising a core period and an extension period, results of the core period were considered in the main analyses. To test the stability of the results, we performed a sensitivity analysis by including the results of the extension periods of the respective trials, provided that double-blind treatment was continued unchanged during the extension period. If treatment changed during the extension period of a trial, only results from the core period were considered. Additional sensitivity analyses excluded studies with a high risk of bias, studies with a treatment duration < 52 weeks and those not designed as cardiovascular outcome trials. Data on different dosages of active treatments and/or comparators were pooled for each study. Study arms including more than one active treatment (= combination therapy) were excluded from endpoint analyses. All *p* values were two-tailed with the statistical significance arbitrarily set at < 0.05.

## Results

### Literature search

The search strategy yielded 73 eligible records reporting on 64 trials [4–6, 22–87]. For three trials (NCT02681094, NCT02630706, NCT00736879), results were not published in a peer-reviewed journal but open to public at www.clinicaltrials.gov. Information on study design and results were thus extracted from www.clinicaltrials.gov. The flowchart of the study selection process is shown in eFig. 1. Agreement between reviewers was excellent (*κ* = 0.935, 95% CI 0.891–0.980).

**Fig. 1:**
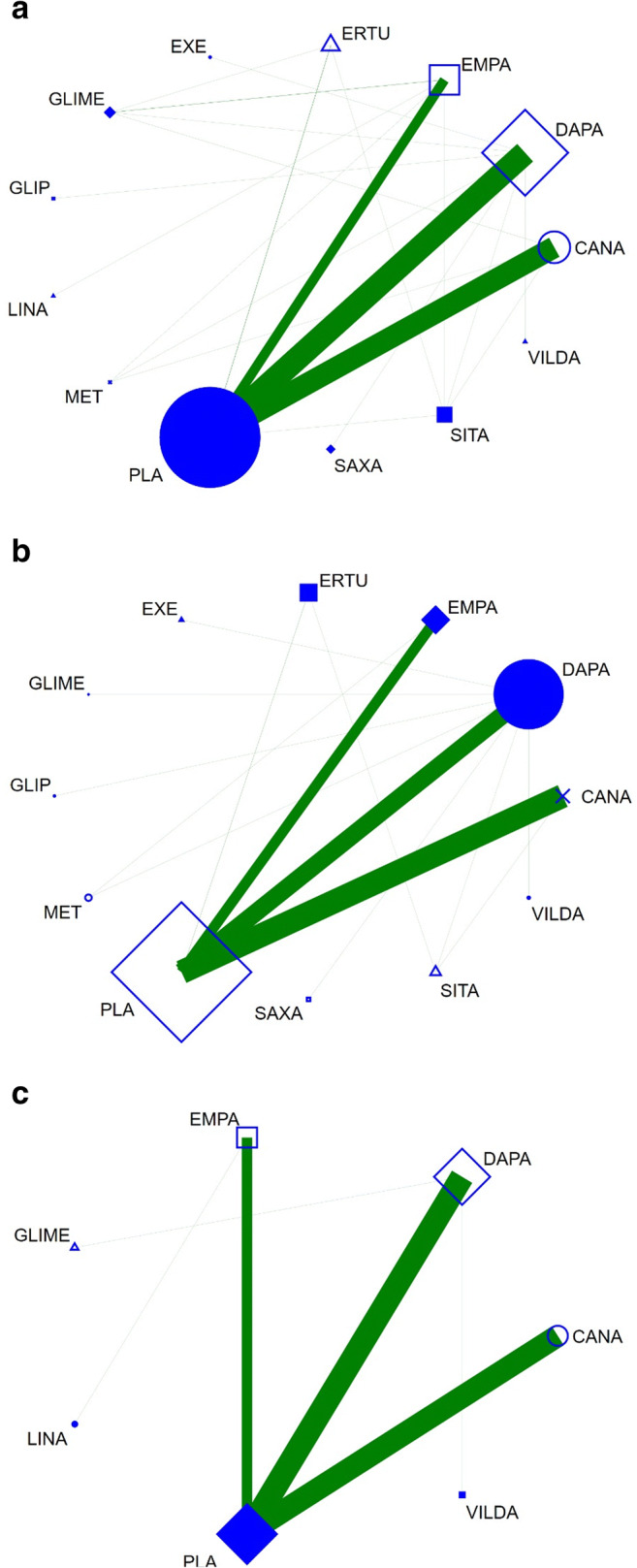
Network plots with respect to **a** all-cause mortality, **b** cardiovascular mortality, and **c** worsening HF. Legend: CANA, canagliflozin; DAPA, dapagliflozin; EMPA, empagliflozin; ERTU, ertugliflozin; EXE, exenatide; GLIME, glimepiride; GLIP, glipizide; LINA, linagliptin; MET, metformin; PLA, placebo; SAXA, saxagliptin; SITA, sitagliptin; VILDA, vildagliptin. Nodes represent the interventions of interest and edges represent available direct comparisons between pairs of interventions. Nodes and edges are weighted according to the number of studies including the respective interventions. Coloured edges are employed to present the risk of bias for each direct comparison in the network, with green, yellow and red colours being used to denote pairwise meta-analyses of low, unclear and high risk of bias

No trials directly compared two different SGLT2i. A total of 44 trials compared SGLT2i with placebo, and 18 trials compared SGLT2i with other active treatments. Two trials compared SGLT2i with both placebo and another active treatment. Canagliflozin was studied in 14 trials (*n* = 22,220 patients), whereas dapagliflozin was studied in 30 trials (*n* = 31,863 patients). Thirteen trials including 15,716 patients investigated the use of empagliflozin, and seven trials studied ertugliflozin (*n* = 5074 patients). The corresponding network plots detailing active treatments and endpoints reported are shown in Fig. 1a–c. All but one were multicentre, parallel-group trials and the mean treatment duration of the core trials was 40 weeks. Fifteen trials comprised a core period and an extension period, in which double-blind treatment was continued unchanged. The mean study duration including extension periods was 52 weeks. In total, the 64 trials reported data from 74,874 patients. Of these, 3155 patients were randomised to a combination treatment of more than one study drug and were therefore excluded from endpoint analyses. Outcome data were thus analysed from 71,719 patients. For study characteristics of trials included in the present NMA, please refer to Table 1.

**Table 1:** Baseline characteristics of included studies

Study	NCT number	Year	Sponsor	Active treatment	Comparator	Centres (*n*)	Patients (*n*)	Treatment duration (weeks)	
								Core period	Extension period
Bailey [22]	NCT00528879	2010	Bristol-Myers Squibb, AstraZeneca	Dapagliflozin	Placebo	80	546	24	n.a.
Bailey [23]	n.a.	2012	Bristol-Myers Squibb, AstraZeneca	Dapagliflozin	Placebo	63	282	24	n.a.
Barnett [24]	NCT01164501	2014	Boehringer Ingelheim, Eli Lilly	Empagliflozin	Placebo	127	738	52	n.a.
Bode [25]	NCT01106651	2013	Janssen Research & Development, LLC	Canagliflozin	Placebo	90	716	26	n.a.
Bolinder [26, 27]	NCT00855166	2012, 2014	Bristol-Myers Squibb, AstraZeneca	Dapagliflozin	Placebo	40	182	24	78
Cefalu [28]	NCT00968812	2013	Janssen Research & Development, LLC	Canagliflozin	Glimepiride	157	1450	52	n.a.
Cefalu [29]	NCT01031680	2015	Bristol-Myers Squibb, AstraZeneca	Dapagliflozin	Placebo	141	922	24	28
Dagogo-Jack [30]	NCT02036515	2018	Merck & Co., Inc.	Ertugliflozin	Placebo	104	462	26	26
DeFronzo [31]	NCT01422876	2015	Boehringer Ingelheim, Eli Lilly	Empagliflozin	Linagliptin	197	686 [413]	24	28
Ferdinand [32]	NCT02182830	2019	Boehringer Ingelheim, Eli Lilly	Empagliflozin	Placebo	92	157	24	n.a.
Ferrannini [33]	NCT00528372	2010	Bristol-Myers Squibb, AstraZeneca	Dapagliflozin	Placebo	85	559	24	n.a.
Fioretto [34]	NCT02413398	2018	AstraZeneca, National Institutes of Health	Dapagliflozin	Placebo	88	321	24	n.a.
Forst [35]	NCT01106690	2014	Janssen Research & Development, LLC	Canagliflozin	Placebo	74	342	26	n.a.^a^
Frias [36], Jabbour [37]	NCT02229396	2016, 2018	AstraZeneca	Dapagliflozin	Exenatide	134	694 [463]	28	25
Grunberger [38]	NCT01986855	2018	Merck Sharp & Dohme Corp., Pfizer Inc.	Ertugliflozin	Placebo	121	467	52	n.a.
Hadjadj [39]	NCT01719003	2016	Boehringer Ingelheim, Eli Lilly	Empagliflozin	Metformin	190	1364 [702]	24	n.a.
Haring [40]	NCT01159600	2013	Boehringer Ingelheim	Empagliflozin	Placebo	148	669	24	n.a.
Haring [41]	NCT01159600	2014	Boehringer Ingelheim	Empagliflozin	Placebo	148	637	24	n.a.
Henry [42]	NCT00643851	2012	Bristol-Myers Squibb, AstraZeneca	Dapagliflozin	Metformin	105	598 [404]	24	n.a.
Henry [42]	NCT00859898	2012	Bristol-Myers Squibb, AstraZeneca	Dapagliflozin	Metformin	131	638 [427]	24	n.a.
Hollander [43, 44]	NCT01999218	2018	Merck & Co., Inc.	Ertugliflozin	Glimepiride	232	1325	52	52
Inagaki [45]	NCT01413204	2014	Mitsubishi Tanabe Pharma Corporation	Canagliflozin	Placebo	5	272	24	n.a.
Jabbour [46]	NCT00984867	2014	Bristol-Myers Squibb, AstraZeneca	Dapagliflozin	Placebo	88	451	24	24
Ji [47]	NCT01095653	2014	Bristol-Myers Squibb, AstraZeneca	Dapagliflozin	Placebo	40	393	24	n.a.
Kadowaki [48]	NCT02354235	2017	Mitsubishi Tanabe Pharma Corporation	Canagliflozin	Placebo	3	138	24	n.a.
Kaku [49]	NCT01294423	2014	Bristol-Myers Squibb, AstraZeneca	Dapagliflozin	Placebo	27	261	24	n.a.
Kohan [50]	NCT00663260	2014	Bristol-Myers Squibb, AstraZeneca	Dapagliflozin	Placebo	111	252	24	70
Kovacs [51, 52]	NCT01210001	2014, 2015	Boehringer Ingelheim	Empagliflozin	Placebo	69	498	24	52
Lavalle-Gonzalez [53]	NCT01106677	2013	Janssen Research & Development, LLC	Canagliflozin	Placebo	169	1284	26	n.a.
Leiter [54]	NCT01042977	2014	Bristol-Myers Squibb, AstraZeneca	Dapagliflozin	Placebo	173	965	24	28
Lewin [55]	NCT01422876	2015	Boehringer Ingelheim, Eli Lilly	Empagliflozin	Linagliptin	197	677 [405]	52	n.a.
Mathieu [56, 57]	NCT01646320	2015, 2016	Bristol-Myers Squibb, AstraZeneca	Dapagliflozin	Placebo	67	320	24	26
Matthaei [58]	NCT01392677	2015	Bristol-Myers Squibb, AstraZeneca	Dapagliflozin	Placebo	46	218	24	n.a.
Müller-Wieland [59]	NCT02471404	2018	AstraZeneca	Dapagliflozin	Glimepiride	194	939 [627]	52	n.a.
Nauck [60]	NCT00660907	2011	Bristol-Myers Squibb, AstraZeneca	Dapagliflozin	Glipizide	95	814	52	n.a.
Neal [4]	NCT01032629, NCT01989754	2017	Janssen Research & Development, LLC	Canagliflozin	Placebo	667	10,142	188	n.a.
Perkovic [61]	NCT02065791	2019	Janssen Research & Development, LLC	Canagliflozin	Placebo	690	4397	136	n.a.
Phrommintikul [62]	NCT03178591	2019	Thailand Research Fund, National Science and Technology Development Agency NSTDA	Dapagliflozin	Vildagliptin	1	49	24	n.a.
Pollock [63]	NCT02547935	2019	AstraZeneca	Dapagliflozin	Placebo	116	448 [296]	24	n.a.
Pratley [64]	NCT02099110	2018	Merck & Co., Inc., Pfizer Inc.	Ertugliflozin	Sitagliptin	21	1232 [745]	52	n.a.
Ridderstrale [65, 66]	NCT01167881	2014, 2018	Boehringer Ingelheim, Eli Lilly	Empagliflozin	Glimepiride	173	1545	104	104
Rodbard [67]	NCT02025907	2013	Janssen Research & Development, LLC	Canagliflozin	Placebo	47	216	26	n.a.
Roden [68, 69]	NCT01177813, NCT01289990	2013, 2015	Boehringer Ingelheim, Eli Lilly	Empagliflozin	Sitagliptin, placebo	124	899	24	52
Rosenstock [70]	NCT00683878	2012	Bristol-Myers Squibb, AstraZeneca	Dapagliflozin	Placebo	105	420	48	n.a.
Rosenstock [71]	NCT01606007	2015	Bristol-Myers Squibb, AstraZeneca	Dapagliflozin	Saxagliptin	139	534 [355]	24	n.a.
Rosenstock [72]	NCT01011868	2015	Boehringer Ingelheim, Eli Lilly	Empagliflozin	Placebo	97	494	78	n.a.
Rosenstock [73]	NCT01809327	2016	Janssen Research & Development, LLC	Canagliflozin	Metformin	158	1186 [712]	26	n.a.
Rosenstock [74]	NCT02033889	2018	Merck Sharp & Dohme Corp., Pfizer	Ertugliflozin	Placebo	?	621	26	n.a.^a^
Schernthaner [75]	NCT01137812	2013	Janssen Research & Development, LLC	Canagliflozin	Sitagliptin	140	755	52	n.a.
Scott [76]	NCT02532855	2018	Merck & Co., Inc.	Dapagliflozin	Sitagliptin	185	614	24	n.a.
Softeland [77]	NCT01734785	2017	Boehringer Ingelheim, Eli Lilly	Empagliflozin	Placebo	90	332	24	n.a.
Stenlof [78]	NCT01081834	2013	Janssen Research & Development, LLC	Canagliflozin	Placebo	79	584	26	n.a.^a^
Strojek [79, 80]	NCT00680745	2011, 2014	Bristol-Myers Squibb, AstraZeneca	Dapagliflozin	Placebo	84	596	24	24
Terra [81]	NCT01958671	2017	Pfizer, Inc., Merck & Co., Inc.	Ertugliflozin	Placebo	67	461	26	n.a. ^a^
Wilding [82, 83]	NCT00673231	2012, 2014	Bristol-Myers Squibb, AstraZeneca	Dapagliflozin	Placebo	126	807	24	80
Wilding [84]	NCT01106625	2013	Janssen Research & Development, LLC	Canagliflozin	Placebo	85	469	26	26
Wiviott [5]	NCT01730534	2019	AstraZeneca	Dapagliflozin	Placebo	882	17,160	218	n.a.
Yale [85]	NCT01064414	2013	Janssen Research & Development, LLC	Canagliflozin	Placebo	89	269	26	n.a.
Yang [86]	NCT01095666	2016	Bristol-Myers Squibb, AstraZeneca	Dapagliflozin	Placebo	32	444	24	n.a.
Yang [87]	NCT02096705	2018	AstraZeneca	Dapagliflozin	Placebo	28	272	24	n.a.
Zinman [6]	NCT01131676	2015	Boehringer Ingelheim, Eli Lilly	Empagliflozin	Placebo	590	7020	164	n.a.
AstraZeneca	NCT00736879		Bristol-Myers Squibb, AstraZeneca	Dapagliflozin	Placebo	62	282	24	n.a.
AstraZeneca	NCT02681094		AstraZeneca	Dapagliflozin	Saxagliptin	119	883 [590]	24	n.a.
Merck Sharp & Dohme Corp.	NCT02630706		Merck Sharp & Dohme Corp., Pfizer	Ertugliflozin	Placebo	50	506	26	n.a.

^a^During the extension period of the trial, patients on placebo or active control changed their treatment. Therefore, only the core period of the respective trial was considered for analyses. *n.a.*, not available. Numbers in brackets show the number of patients available for endpoint analyses (if different to the total number of patients included in the respective trial)

### Patient characteristics

Patients were on average between 52 and 69 years old and baseline HbA1c varied between 7.2% and 9.3%. The majority of patients had preserved renal function. The prevalence of cardiovascular disease was reported in 15 trials and varied between 26.1% and 100%, totalling 26,360 patients. A total of 7534 patients from 14 trials was treatment-naïve, whereas 67,340 patients received background treatment for diabetes with OADs and/or insulin. For details, please see eTable 1.

### Risk of bias

The overall risk of bias was low. With respect to the individual items of the risk of bias assessment (eFig. 2), the majority of studies provided adequate random sequence generation with good group balance at baseline. All-cause mortality could be retrieved for all but one trial, whereas cardiovascular mortality was reported in 46 (71.9%) trials (*n* = 59,168 patients). Data on HF outcomes were available for 42,683 patients included in 12 trials. There was no systematic association between type or size of the trial or the publication date and any pattern of missing endpoint information. The comparison adjusted funnel plot for all-cause mortality (eFig. 3) was symmetrical, suggesting the absence of small-study effects and publication bias.

### Outcomes

For all endpoints including the respective outcome numbers per trial arm, please refer to eTable 2.

#### All-cause mortality

**Fig. 2:**
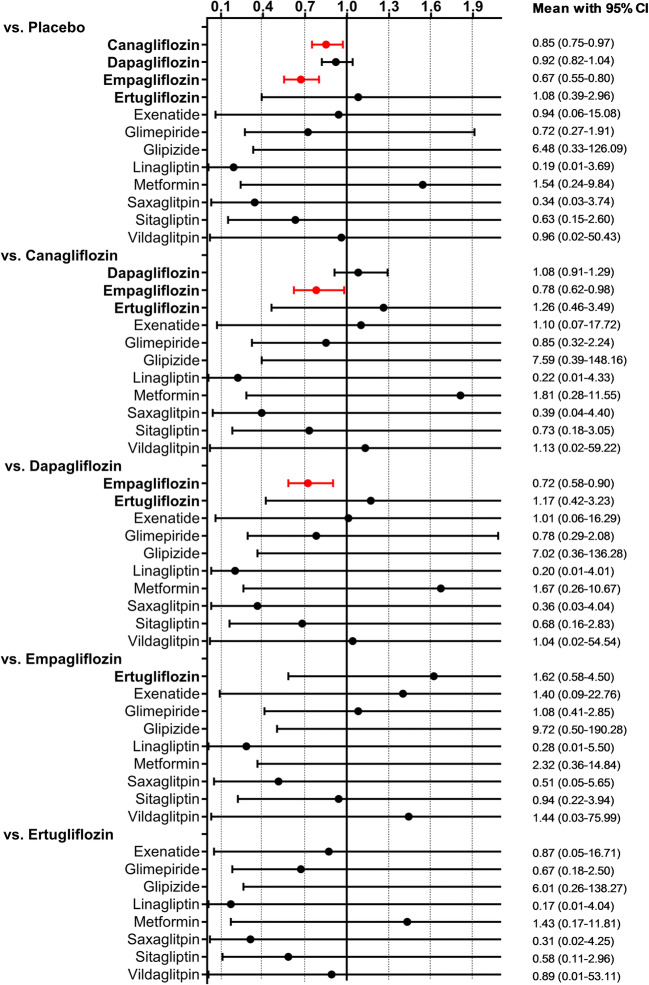
Predictive interval plot for all-cause mortality. Legend: The predictive interval plot represents a forest plot of the joint estimated summary effects from both direct and indirect comparisons along with their confidence intervals. Significant results are shown in read colour

The predictive interval plot summarizing the relative mean effects along with the impact of heterogeneity on the respective confidence interval (= the predictive interval) of each (network) comparison is shown in Fig. 2. Canagliflozin, dapagliflozin and empagliflozin all had a beneficial effect on all-cause mortality compared with placebo. In head-to-head comparisons, the analysis suggests that empagliflozin is superior to both canagliflozin and dapagliflozin. No other head-to-head comparison of any pair of treatments (including non-SGLT2 treatments) found a significant difference between agents, though for most of these comparisons, the 95% CI was wide. SUCRA values are presented in Table 2. The graphical display of the ranking based on the SUCRA values is shown in eFig. 4. The inconsistency within the respective closed loops for each comparison was overall low (eFig. 5) and did not reach statistical significance for any of the loops.

**Table 2:** Surface under the cumulative ranking curve (SUCRA) values for all endpoints

SUCRA	All-cause mortality	Cardiovascular mortality	Worsening HF
Canagliflozin	0.519	0.533	0.754
Dapagliflozin	0.437	0.414	0.537
Empagliflozin	0.684	0.697	0.677
Ertugliflozin	0.385	0.659	n.a.
Placebo	0.335	0.374	0.285

*HF*, heart failure; *n.a.*, not available. SUCRA is a transformation of the mean rank that accounts both for the location and the variance of all relative treatment effects. SUCRA would be 1 when a treatment is certain to be the best and 0 when a treatment is certain to be the worst [19]

#### Cardiovascular mortality

**Fig. 3:**
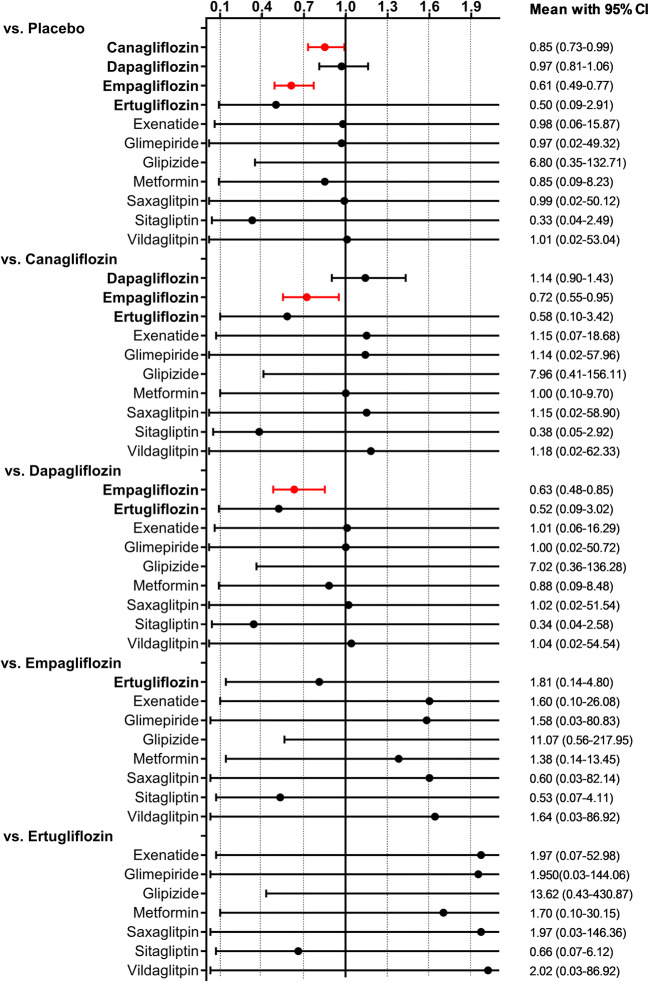
Predictive interval plot for cardiovascular mortality. Legend: The predictive interval plot represents a forest plot of the joint estimated summary effects from both direct and indirect comparisons along with their confidence intervals. Significant results are shown in red colour

The predictive interval plot (Fig. 3) showed that empagliflozin was again superior to placebo, canagliflozin and dapagliflozin in reducing cardiovascular mortality. Canagliflozin also reduced cardiovascular mortality compared with placebo. No other head-to-head comparison of any pair of treatments (including non-SGLT2 treatments) found a significant difference between agents, though again for most of these comparisons, the 95% CI was wide. SUCRA values are presented in Table 2. The graphical display of the ranking based on the SUCRA values is shown in eFig. 6. The inconsistency within the respective closed loops for each comparison was overall low (eFig. 7) and again did not reach statistical significance for any of the loops.

#### Worsening HF

**Fig. 4:**
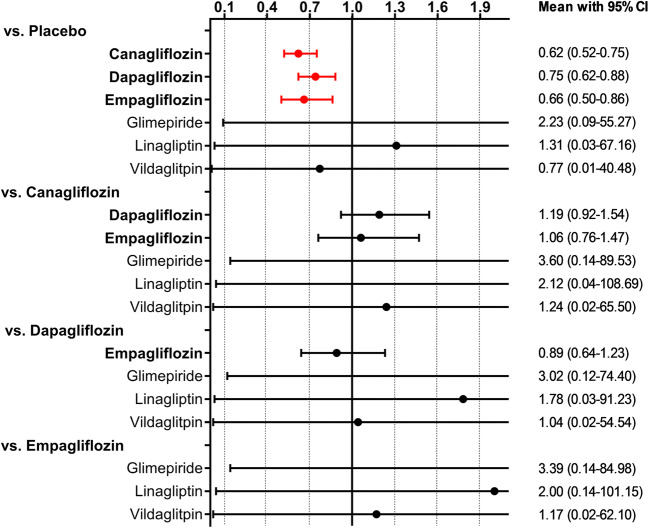
Predictive interval plot for worsening HF. Legend: The predictive interval plot represents a forest plot of the joint estimated summary effects from both direct and indirect comparisons along with their confidence intervals. Significant results are shown in red colour

The predictive interval plot (Fig. 4) showed that canagliflozin, dapagliflozin and empagliflozin all reduced the endpoint of worsening HF when compared with placebo. There were no further significant differences in HF outcomes between individual SGLT2i. SUCRA values are presented in Table 2. The graphical display of the ranking based on the SUCRA values is shown in eFig. 8. No closed loops were formed and consequently no inconsistency could be derived.

### Sensitivity analyses

Sensitivity analyses essentially confirmed our main results. When we included the results of study extension periods to the outcome analyses, empagliflozin was again more effective in reducing all-cause and cardiovascular mortality than all other agents, while there was no difference between the individual SGLT2i in reducing worsening HF (eFig. 9, 10 and 11). Results were similar after excluding trials with a treatment duration < 52 weeks (eFig. 12, 13 and 14) or when restricting our analyses to patients included in cardiovascular outcome trials (*n* = 38,719; eFig. 15, 16 and 17). As we did not identify any trials with a high risk of bias, the corresponding sensitivity analysis was not appropriate.

## Discussion

SGLT2i belong to a new class of OAD that confer benefits on cardiovascular endpoints in patients with T2D. To date, there is no randomised controlled trial (RCT) or retrospective head-to-head comparison of any available SGLT2i. NMA is an increasingly popular tool for comparative effectiveness research. The integration of direct (head-to-head) and indirect (transitively derived via a common comparator) evidence allows for comparisons that otherwise elude conventional (aggregate) analysis while increasing precision in the estimates along the way. The present analysis is thus the first to provide evidence of the comparative cardiovascular effects of different SGLT2i in patients with T2D.

In a comprehensive analysis of almost 75,000 patients derived from 64 trials, we found that while empagliflozin, canagliflozin and dapagliflozin reduce all-cause mortality compared with placebo, empagliflozin appears more effective than the latter two. These results were essentially mirrored for cardiovascular mortality, while all three appear of similar efficacy with respect to worsening HF. Ertugliflozin had no effect on any of the three endpoints investigated.

The mortality advantage of empagliflozin reflects the results of four recently published large-scale placebo-controlled cardiovascular outcome trials, since these trials contribute more than 90% of mortality events to the present analysis. In the EMPA-REG-OUTCOME trial, empagliflozin significantly reduced all-cause and cardiovascular mortality in 7020 patients with T2D at high cardiovascular risk [6]. In contrast, dapagliflozin had a neutral effect on survival in 17,160 patients included in the DECLARE-TIMI 58 trial [5]. Similarly, canagliflozin did not affect overall survival or cardiovascular death both in 10,142 patients enrolled in the CANVAS programme and in 4401 patients enrolled in the CREDENCE trial [4, 61], though in all cases, the 95% CI of the effect measure just touched the null-effect line.

Our findings may reflect features of trial designs or actual differences between the agents. Although molecules of dapagliflozin and canagliflozin are very similar to those of empagliflozin, small differences in the molecular structure can potentially lead to critical differences. For example, the molecular differences between the hormones testosterone and estradiol are substantially smaller than the differences between the empagliflozin molecule and the other two members of the class [88]. However, individual SGLT2i share their mode of action as well as important pharmacological characteristics including bioavailability, receptor selectivity, metabolism, elimination half-life and excretion [89, 90]. In addition, they have comparable effects on blood glucose, body weight and blood pressure, which are the suggested mediators of the anti-atherosclerotic effects of SGLT2i.

The difference in survival benefit between individual SGLT2i may potentially be explained by differences in trial populations. For example, the number of patients with established atherosclerotic cardiovascular disease in EMPA-REG-OUTCOME was significantly higher than in the other trials. The mortality rate in the placebo group of the EMPA-REG-OUTCOME trial was higher than in the other SGLT2i cardiovascular outcome trials, highlighting the differences between populations. An additional factor is that the number of patients with concomitant chronic kidney disease varied between trials. As patients with impaired renal function may gain a greater benefit from SGLT2i therapy, exclusion of these patients from the DECLARE-TIMI 58 trial may have limited mortality benefits [91].

To date, data on the cardiovascular effects of ertugliflozin are scarce. The present NMA includes seven trials totalling 4740 patients treated with ertugliflozin; however, these trials reported only 17 deaths. Due to the low number of events, mortality analyses result in wide confidence intervals and should therefore be interpreted with caution. The cardiovascular efficacy and safety of ertugliflozin in patients with T2D is currently being evaluated in the VERTIS-CV trial. The trial completed enrolment in 2017 and the results are expected to be published in 2020 [92].

The present NMA shows a clear reduction in HF events with canagliflozin, dapagliflozin and empagliflozin, with no significant difference between individual SGLT2i. Again, these results are mainly driven by the four large-scale cardiovascular outcome trials, which reported a relative 30–40% risk reduction for worsening HF for each agent [4–6, 61]. Notably, the benefit was independent of baseline cardiovascular risk or a history of HF [93–95]. The benefits with SGLT2i for HF outcomes may be secondary to a reduction in circulating volume and other haemodynamic effects with a reduction of myocardial loading [93–95]. Natriuresis [96], systemic blood pressure lowering [97], modification of the intrarenal renin angiotensin axis [98] and reduction in arterial stiffness [99] may all contribute to the protection afforded [94]. These effects have been reported for all the different SGLT2i, consistent with the comparable HF efficacy of individual SGLT2i.

In the present NMA, no significant differences in mortality or HF efficacy were found when comparing individual SGLT2i to other active treatments. This contrasts to two recently published meta-analyses in which the use of SGLT2 inhibitors was associated with lower mortality and a lower risk of HF compared with dipeptidyl peptidase 4 inhibitors [100, 101]. The meta-analyses, however, compared classes of drugs, whereas the present NMA presents comparisons of individual agents. As the number of events included in each analysis is low, comparisons of individual SGLT2i with other active treatments need to be interpreted with caution.

## Limitations

The present NMA includes all the available evidence regarding the effects of SGLT2i on commonly accepted endpoints in patients with T2D. It deliberately excludes the recently published DAPA-HF trial [102]. This is because all trials (except DAPA-HF) included T2D patients—of whom some had chronic HF—while DAPA-HF included only chronic HF patients—of whom some had T2D. This would substantially skew baseline characteristics between DAPA-HF and all other studies. The ensuing violation of the transitivity assumption would thus invalidate the entire NMA. Several other potential study limitations should be considered.

First, most trials in the present NMA included a relatively small number of patients, with four trials contributing almost half of the study population.

Second, the mean follow-up duration of the core trials was 40 weeks, which limits mortality analyses. However, our results were confirmed in a sensitivity analysis restricted to trials with a treatment duration of at least 52 weeks.

Third, the majority of studies included were not designed as cardiovascular outcome trials. They were therefore not powered to detect differences in survival between active treatments and comparators. However, aggregation of individual trial data in a (network) meta-analysis is an appropriate tool to increase the power and validity of individual study results. In addition, we confirmed the results of our NMA in a sensitivity analysis that excluded non-cardiovascular outcome trials.

Fourth, baseline cardiovascular risk—if reported at all—varied substantially between trials, with a significantly higher number of patients with established atherosclerotic cardiovascular disease included in empagliflozin trials. As patients at high cardiovascular risk may gain a greater benefit from SGLT2i therapy, differences between trial populations may have biased the results. In addition, differences in background anti-diabetic and/or cardiovascular treatment may have affected the number of cardiovascular endpoints.

Fifth, statistically significant results from a (network) meta-analysis do not necessarily imply clinically meaningful differences in efficacy. The findings of the present study should therefore be interpreted cautiously.

## Conclusion

We found similar reductions in worsening HF with empagliflozin, canagliflozin and dapagliflozin. However, empagliflozin was associated with a greater reduction in all-cause and cardiovascular mortality. Due to the low number of events reported from ertugliflozin trials, no reliable conclusions on cardiovascular outcomes may be drawn from ertugliflozin analyses. Although the differences in the efficacy of individual SGLT2i might reflect different trial designs, clinicians may prefer empagliflozin over other SGLT2i until more evidence on the comparative efficacy of SGLT2i is available.

## Electronic supplementary material

ESM 1 (PDF 2994 kb)
