# Supplementary material for: Comparative efficacy of sodium-glucose cotransporter-2 inhibitors (SGLT2i) for cardiovascular outcomes in type 2 diabetes: a systematic review and network meta-analysis of randomised controlled trials
Source: Heart Fail Rev. 2020 Apr 20;26(6):1421–35. doi: 10.1007/s10741-020-09954-8 (PMC8510986; doi:10.1007/s10741-020-09954-8)
Supplement: Supplementary file 1 — (PDF 2994 kb) [file 10741_2020_9954_MOESM1_ESM.pdf]

Supplementary material

Supplement to: Täger et al. Comparative efficacy of sodium-glucose cotransporter-2 inhibitors (SGLT2i) for cardiovascular outcomes in type 2 diabetes: a systematic review and network meta-analysis of randomised controlled trials**.** Heart Failure Reviews 2020.

Corresponding author:

Prof. Dr. med. Lutz Frankenstein

University Hospital Heidelberg

Department of cardiology, angiology, and pulmology

Im Neuenheimer Feld 410

69120 Heidelberg, Germany

Phone: +49 6221 56 38995

Email: lutz.frankenstein@med.uni-heidelberg.de

Appendix

Search Algorithms 3
Supplementary Figures 4
eFig. 1 4
eFig. 2 5
eFig. 3 6
eFig. 4 7
eFig. 5 8
eFig. 6 9
eFig. 7 10
eFig. 8 11
eFig. 9 12
eFig. 10 13
eFig. 11 14
eFig. 12 15
eFig. 13 16
eFig. 14 17
eFig. 15 18
eFig. 16 19
eFig. 17 20
Supplementary Tables 21
eTable 1 21
eTable 2 26
Supplementary References 31

Search Algorithms

Pubmed and Cochrane Central Register of Controlled Trials search:

("type 2 diabetes mellitus" OR "T2D" OR "diabetes mellitus type 2" OR "type 2 diabetes" OR "diabetes type 2") AND ("SGLT2 inhibitor*" OR "SGLT2i" OR "Empagliflozin" OR "Dapagliflozin" OR "Canagliflozin" OR "Ertugliflozin") AND ("cardiovascular outcome" OR "myocardial infarction" OR "stroke" OR "heart failure" OR "MACE" OR "major adverse cardiac events" OR "major adverse cardiovascular events" OR "death") AND (trial OR random* OR controlled) NOT review

www.clinicaltrials.gov search

Condition or disease: type 2 diabetes mellitus OR diabetes mellitus type 2 OR type 2 diabetes OR diabetes type 2 OR T2D OR T2DM

Other terms: Empagliflozin OR Canagliflozin OR Dapagliflozin OR Ertugliflozin

Applied Filters: Completed, adult (18–64), older adult (65+), phase 3, phase 4

Supplementary Figures

eFig. 1 Flow chart of study identification and selection

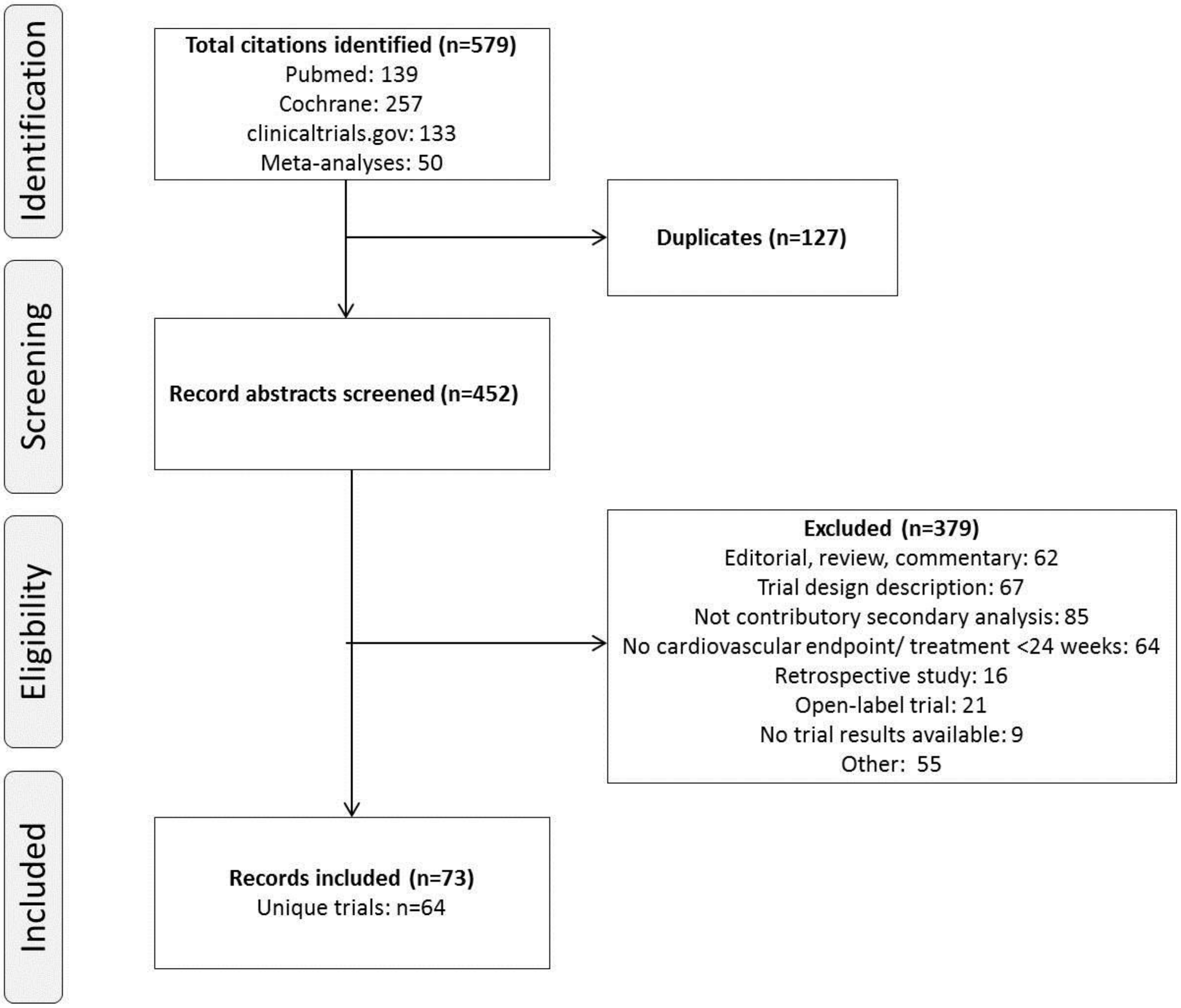

eFig. 2 Individual risk of bias assessment

Study	Random sequence generation (selection bias)	Allocation concealment (selection bias)	Blinding of participants and personnel (performance bias)	Blinding of outcome assessment (detection bias)	Intention-to-treat analysis (attrition bias)	Incomplete outcome data (attrition bias)	Groups balanced at baseline (selection bias)	Selective reporting (reporting bias)	Independent funding	Trials stopped early	Prospective clinical trial registration
Bailey [22]	✓	✓	✓	✓	✓	✓	✓	✓	✗	✓	✓
Bailey [23]	✓	✓	✓	✓	✓	✓	✓	✓	✗	✓	✗
Barnett [24]	✓	✓	✓	✓	✓	✓	✓	!	✗	✓	✓
Bode [25]	✓	✓	✓	✓	✓	✓	✓	✓	✗	✓	✓
Bolinder [26,27]	✓	✓	✓	✓	✓	✓	✓	✓	✗	✓	✓
Cefalu [28]	✓	✓	✓	✓	✓	✓	✓	✓	✗	✓	✓
Cefalu [29]	✓	✓	✓	✓	✓	✓	✓	✓	✗	✓	✓
Dagogo-Jack [30]	✓	✓	✓	✓	✓	✓	✓	✓	✗	✓	✓
DeFronzo [31]	✓	✓	✓	✓	✓	✓	✓	✓	✗	✓	✓
Ferdinand [32]	✓	✓	✓	✓	✓	✓	✓	✓	✗	✓	✓
Ferrannini [33]	✓	✓	✓	✓	✓	✓	✓	✓	✗	✓	✓
Fioretto [34]	✓	✓	✓	✓	✓	✓	✓	✓	✗	✓	✓
Forst [35]	✓	✓	✓	✓	✓	✓	✓	✓	✗	✓	✓
Frias [36], Jabbour [37]	✓	✓	✓	✓	✓	✓	✓	✓	✗	✓	✓
Grunberger [38]	✓	✓	✓	✓	✓	✓	✓	✓	✗	✓	✓
Hadjadj [39]	✓	✓	✓	✓	✓	✓	✓	!	✗	✓	✓
Haring [40]	✓	✓	✓	✓	✓	✓	✓	✓	✗	✓	✓
Haring [41]	✓	✓	✓	✓	✓	✓	✓	✓	✗	✓	✓
Henry [42]	✓	✓	✓	✓	✓	✓	✓	✓	✗	✓	✓
Henry [42]	✓	✓	✓	✓	✓	✓	✓	✓	✗	✓	✓
Hollander [43,44]	✓	✓	✓	✓	✓	✓	✓	✓	✗	✓	✓
Inagaki [45]	✓	✓	✓	✓	✓	✓	✓	✓	✗	✓	✓
Jabbour [46]	✓	✓	✓	✓	✓	✓	✓	✓	✗	✓	✓
Ji [47]	✓	✓	✓	✓	✓	✓	✓	✓	✗	✓	✓
Kadowaki [48]	✓	✓	✓	✓	✓	✓	✓	!	✗	✓	✓
Kaku [49]	✓	✓	✓	✓	✓	✓	✓	✓	✗	✓	✓
Kohan [50]	✓	✓	✓	✓	✓	✓	✓	✓	✗	✓	✓
Kovacs [51,52]	✓	✓	✓	✓	✓	✓	✓	✓	✗	✓	✓
Lavalle-Gonzalez [53]	✓	✓	✓	✓	✓	✓	✓	✓	✗	✓	✓
Leiter [54]	✓	✓	✓	✓	✓	✓	✓	!	✗	✓	✓
Lewin [55]	✓	✓	✓	✓	✓	✓	✓	!	✗	✓	✓
Mathieu [56,57]	✓	✓	✓	✓	✓	✓	✓	✓	✗	✓	✓
Matthaei [58]	✓	✓	✓	✓	✓	✓	✓	✓	✗	✓	✓
Müller-Wieland [59]	✓	✓	✓	✓	✓	✓	✓	!	✗	✓	✓
Nauck [60]	✓	✓	✓	✓	✓	✓	✓	✓	✗	✓	✓
Neal [4]	✓	✓	✓	✓	✓	✓	✓	✓	✗	✓	✓
Perkovic [61]	✓	✓	✓	✓	✓	✓	✓	✓	✗	✓	✓
Phrommintikul [62]	✓	✓	✓	✓	✓	✓	✓	!	✓	✓	✓
Pollock [63]	✓	✓	✓	✓	✓	✓	✓	✓	✗	✓	✓
Pratley [64]	✓	✓	✓	✓	✓	✓	✓	✓	✗	✓	✓
Ridderstrale [65,66]	✓	✓	✓	✓	✓	✓	✓	✓	✗	✓	✓
Rodbard [67]	✓	✓	✓	✓	✓	✓	✓	✓	✗	✓	✓
Roden [68,69]	✓	✓	✓	✓	✓	✓	✓	✓	✗	✓	✓
Rosenstock [70]	✓	✓	✓	✓	✓	✓	✓	✓	✗	✓	✓
Rosenstock [71]	✓	✓	✓	✓	✓	✓	✓	✓	✗	✓	✓
Rosenstock [72]	✓	✓	✓	✓	✓	✓	✓	✓	✗	✓	✓
Rosenstock [73]	✓	✓	✓	✓	✓	✓	✓	✓	✗	✓	✓
Rosenstock [74]	✓	✓	✓	✓	✓	✓	✓	✓	✗	✓	✓
Schernthaner [75]	✓	✓	✓	✓	✓	✓	✓	✓	✗	✓	✓
Scott [76]	✓	✓	✓	✓	✓	✓	✓	✓	✗	✓	✓
Softeland [77]	✓	✓	✓	✓	✓	✓	✓	✓	✗	✓	✓
Stenlof [78]	✓	✓	✓	✓	✓	✓	✓	✓	✗	✓	✓
Strojek [79,80]	✓	✓	✓	✓	✓	✓	✓	✓	✗	✓	✓
Terra [81]	✓	✓	✓	✓	✓	✓	✓	✓	✗	✓	✓
Wilding [82,83]	✓	✓	✓	✓	✓	✓	✓	✓	✗	✓	✓
Wilding [84]	✓	✓	✓	✓	✓	✓	✓	✓	✗	✓	✓
Wiviott [5]	✓	✓	✓	✓	✓	✓	✓	✓	✗	✓	✓
Yale [85]	✓	✓	✓	✓	✓	✓	✓	!	✗	✓	✓
Yang [86]	✓	✓	✓	✓	✓	✓	✓	✓	✗	✓	✓
Yang [87]	✓	✓	✓	✓	✓	✓	✓	✓	✗	✓	✓
Zinman [6]	✓	✓	✓	✓	✓	✓	✓	✓	✗	✓	✓
AstraZeneca	✓	✓	✓	✓	✓	✓	✓	!	✗	✓	✓
AstraZeneca	✓	✓	✓	✓	✓	✓	✓	!	✗	✓	✓
Merck Sharp & Dohme Corp.	✓	✓	✓	✓	✓	✓	✓	!	✗	✓	✓

eFig. 3 Comparison funnel plot (all-cause mortality)

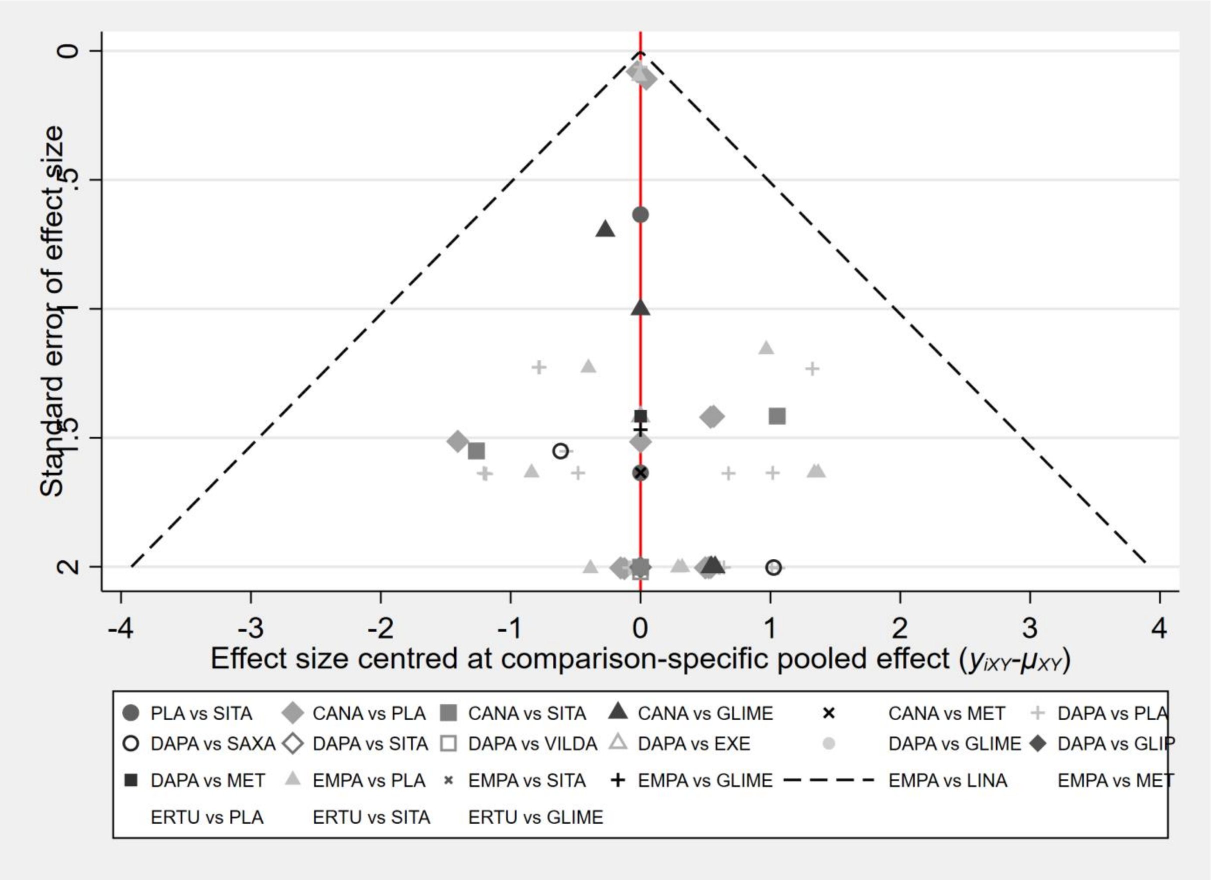

Legend: CANA, canagliflozin; DAPA, dapagliflozin; EMPA, empagliflozin; ERTU, ertugliflozin; EXE, exenatide; GLIME, glimepiride; GLIP, glipizide; LINA, linagliptin; MET, metformin; PLA, placebo; SAXA, saxagliptin; SITA, sitagliptin; VILDA, vildagliptin.

eFig. 4 Graphical ranking of SGLT2i based on SUCRA values (all-cause mortality)

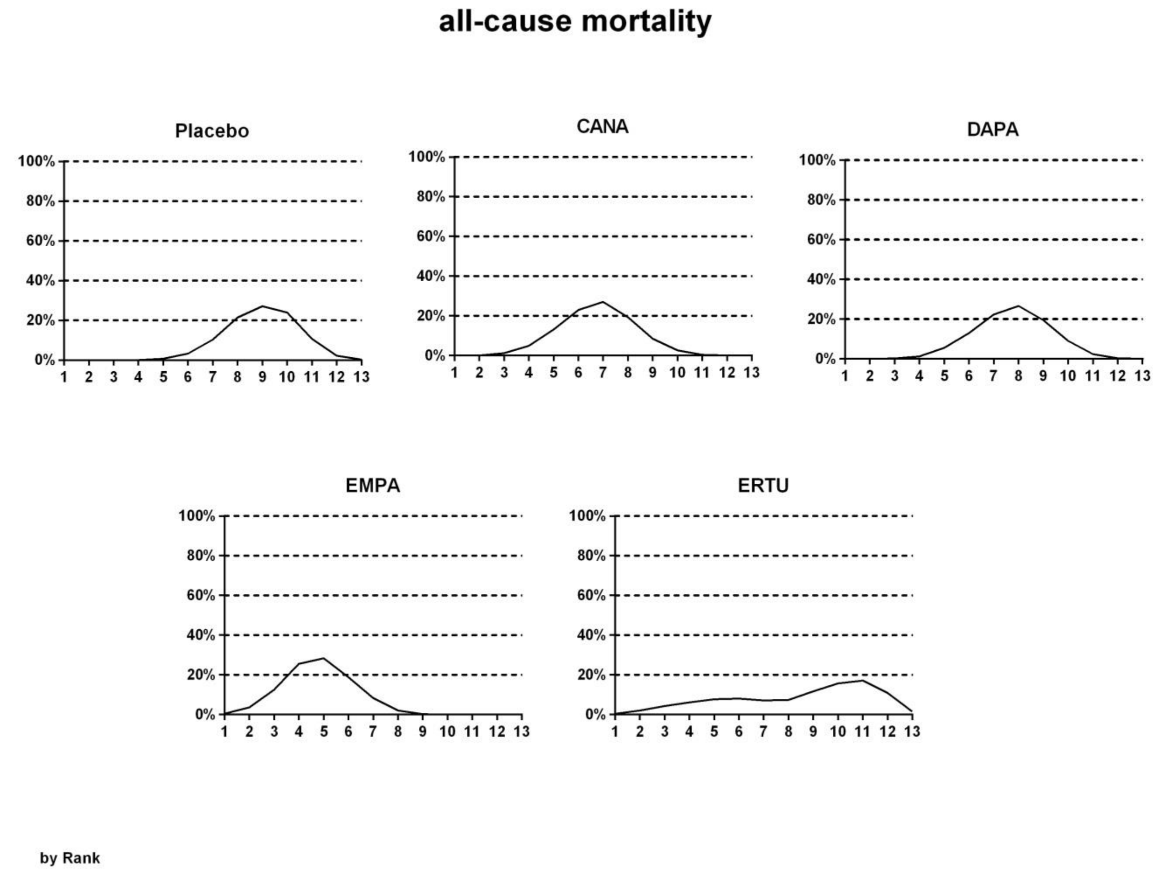

Legend: CANA, canagliflozin; DAPA, dapagliflozin; EMPA, empagliflozin; ERTU, ertugliflozin.

eFig. 5 Plot of inconsistency between direct and various indirect effect for any (same) comparison within closed loops regarding all-cause mortality

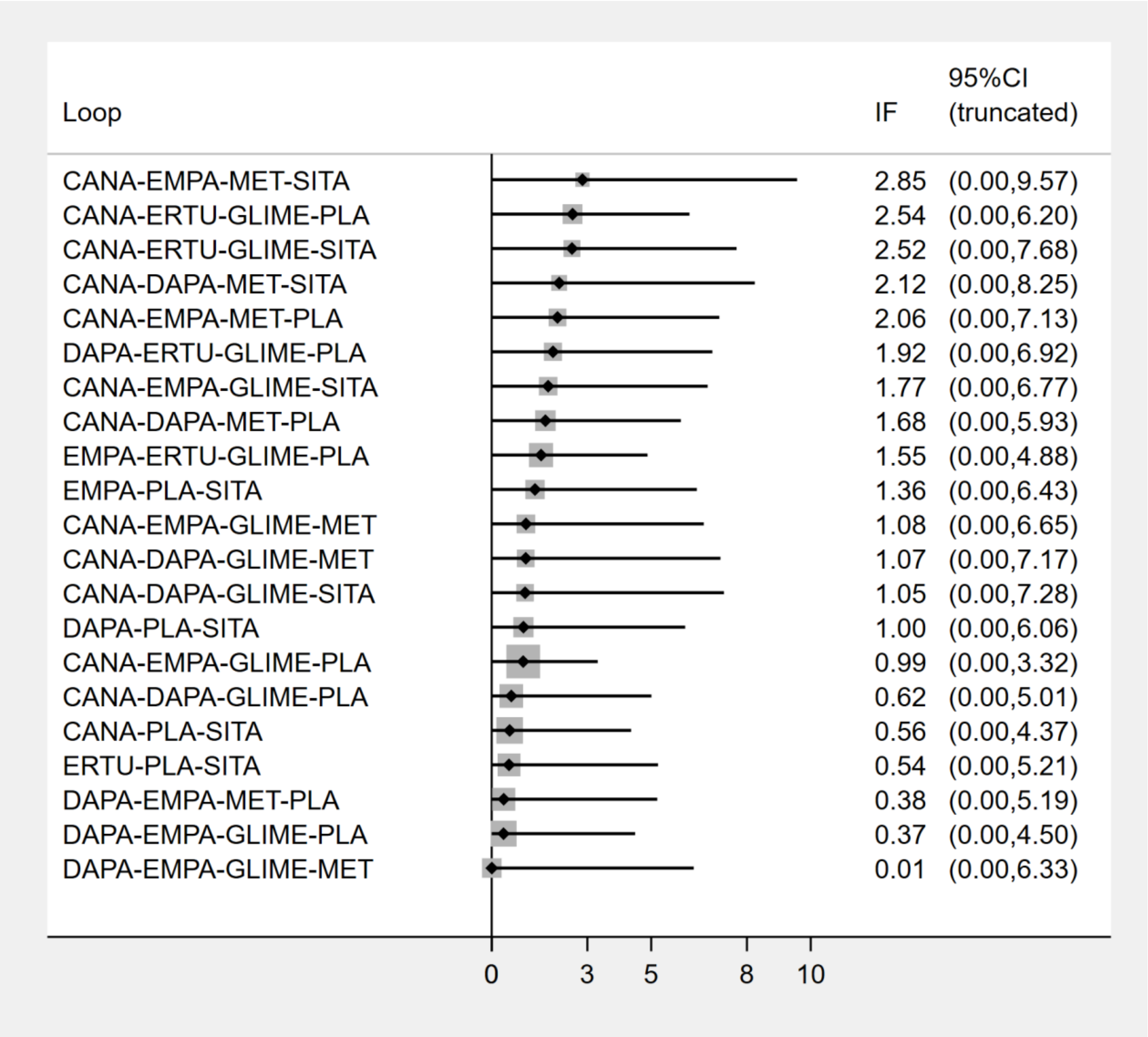

Legend: CANA, canagliflozin; DAPA, dapagliflozin; EMPA, empagliflozin; ERTU, ertugliflozin; EXE, exenatide; GLIME, glimepiride; GLIP, glipizide; IF, inconsistency factor; LINA, linagliptin; MET, metformin; PLA, placebo; SAXA, saxagliptin; SITA, sitagliptin; VILDA, vildagliptin.

eFig. 6 Graphical ranking of SGLT2i based on SUCRA values (cardiovascular mortality)

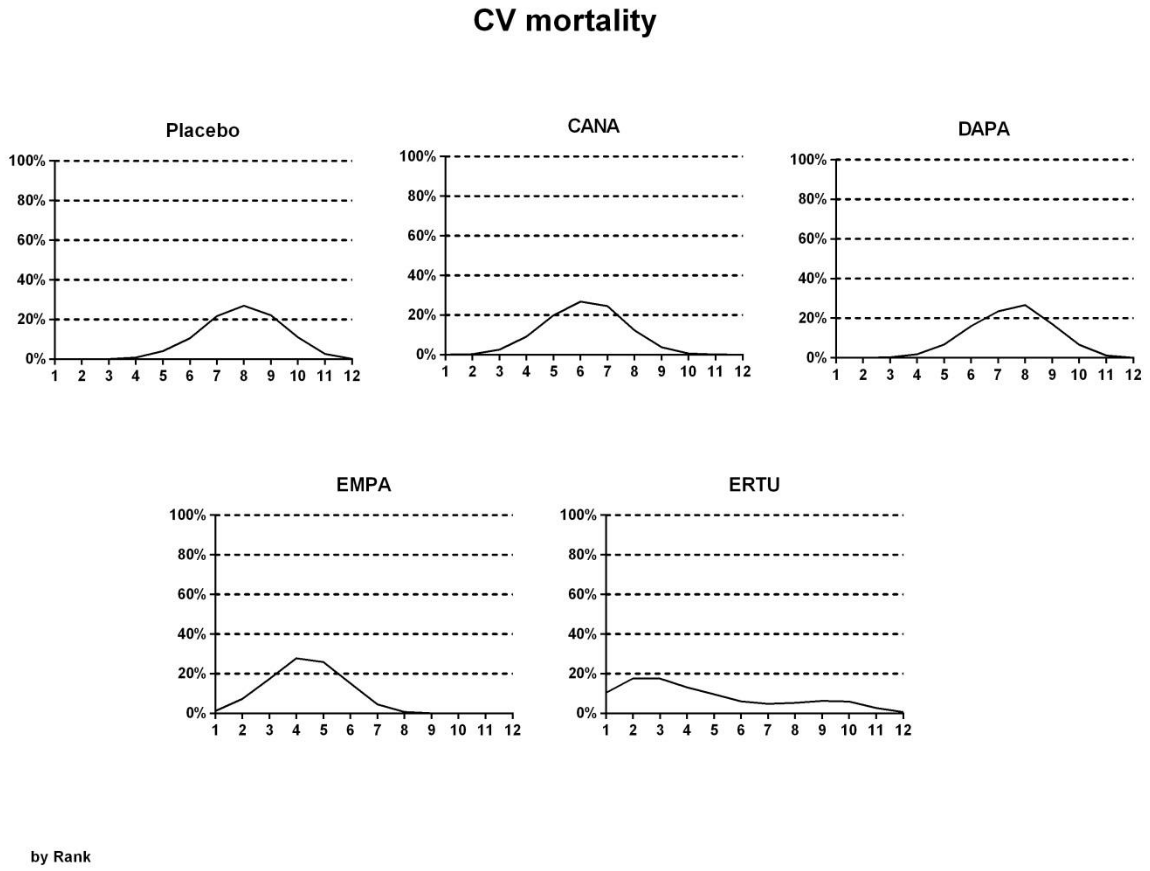

Legend: CANA, canagliflozin; DAPA, dapagliflozin; EMPA, empagliflozin; ERTU, ertugliflozin.

eFig. 7 Plot of inconsistency between direct and various indirect effect for any (same) comparison within closed loops regarding cardiovascular mortality

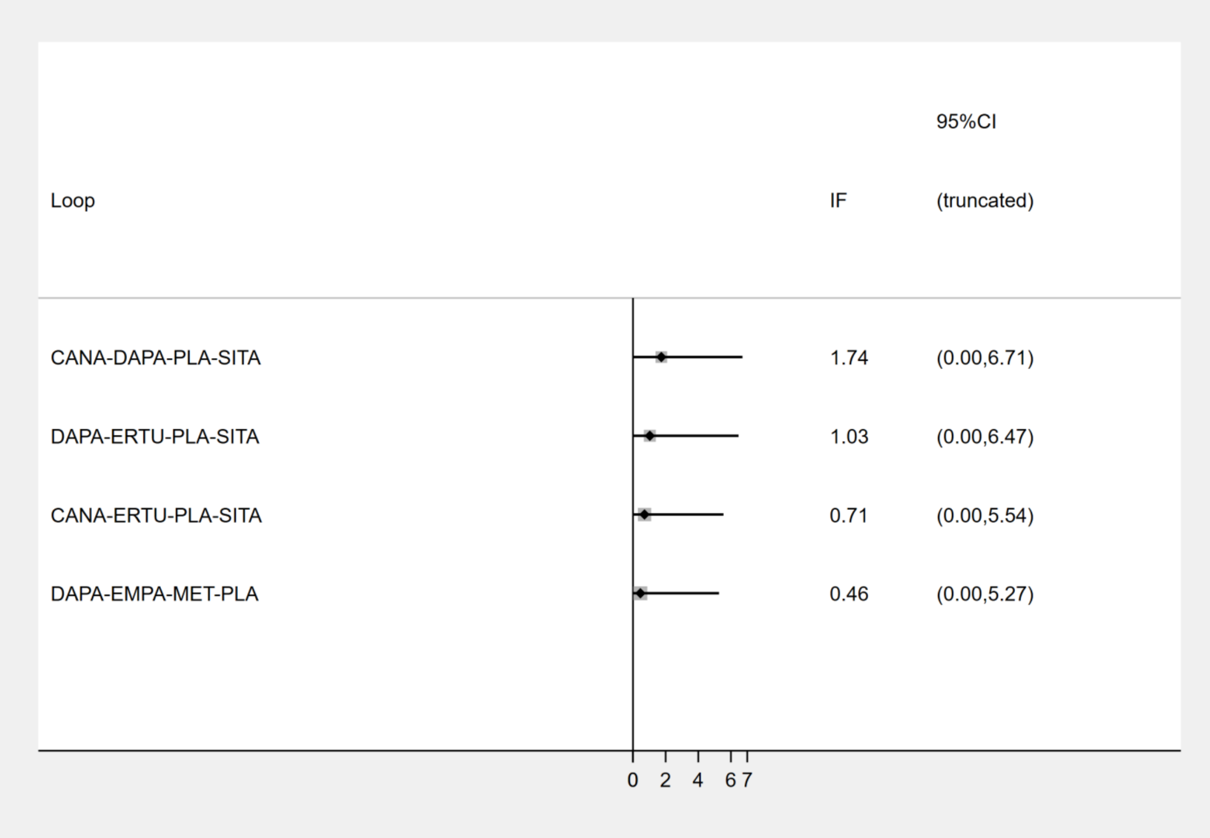

Legend: CANA, canagliflozin; DAPA, dapagliflozin; EMPA, empagliflozin; ERTU, ertugliflozin; IF, inconsistency factor ; MET, metformin; PLA, placebo.

eFig. 8 Graphical ranking of SGLT2i based on SUCRA values (worsening HF)

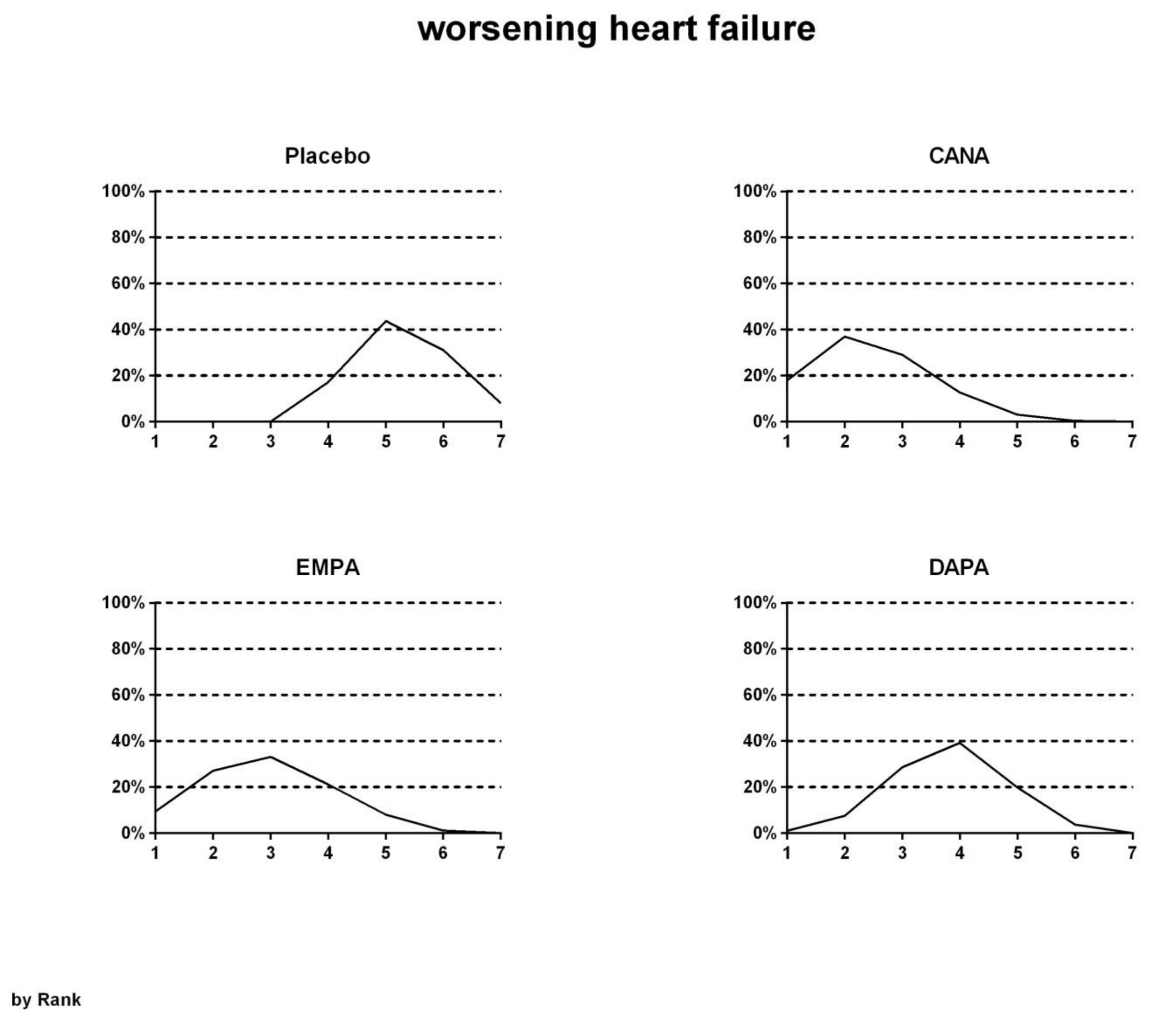

Legend: CANA, canagliflozin; DAPA, dapagliflozin; EMPA, empagliflozin.

eFig. 9 Predictive interval plot for all-cause mortality in sensitivity analyses including extension periods of trials

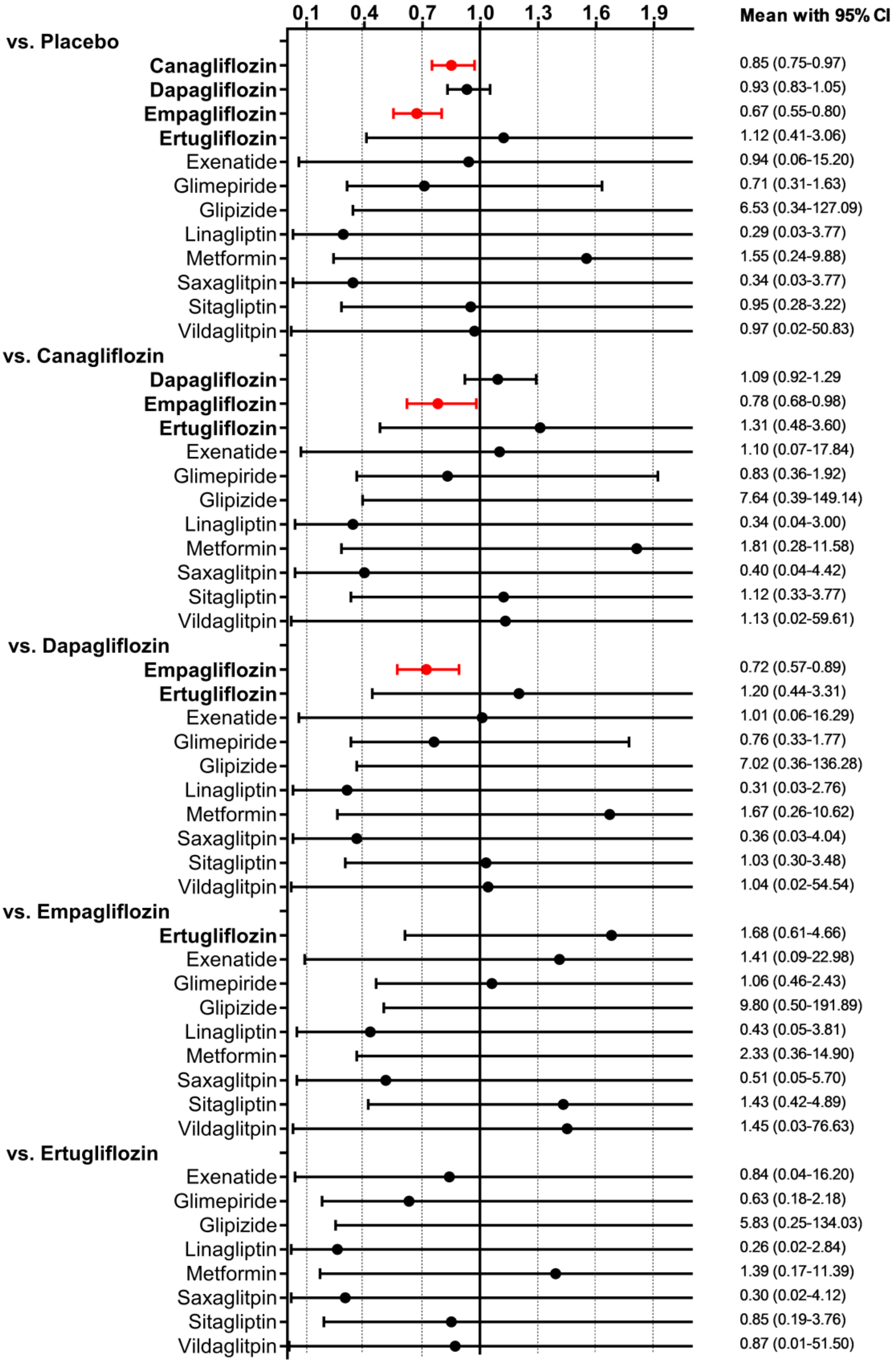

Legend: The predictive interval plot represents a forest plot of the joint estimated summary effects from both direct and indirect comparisons along with their confidence intervals. Significant estimated summary effects are shown in red colour.

eFig. 10 Predictive interval plot for cardiovascular mortality in sensitivity analyses including extension periods of trials

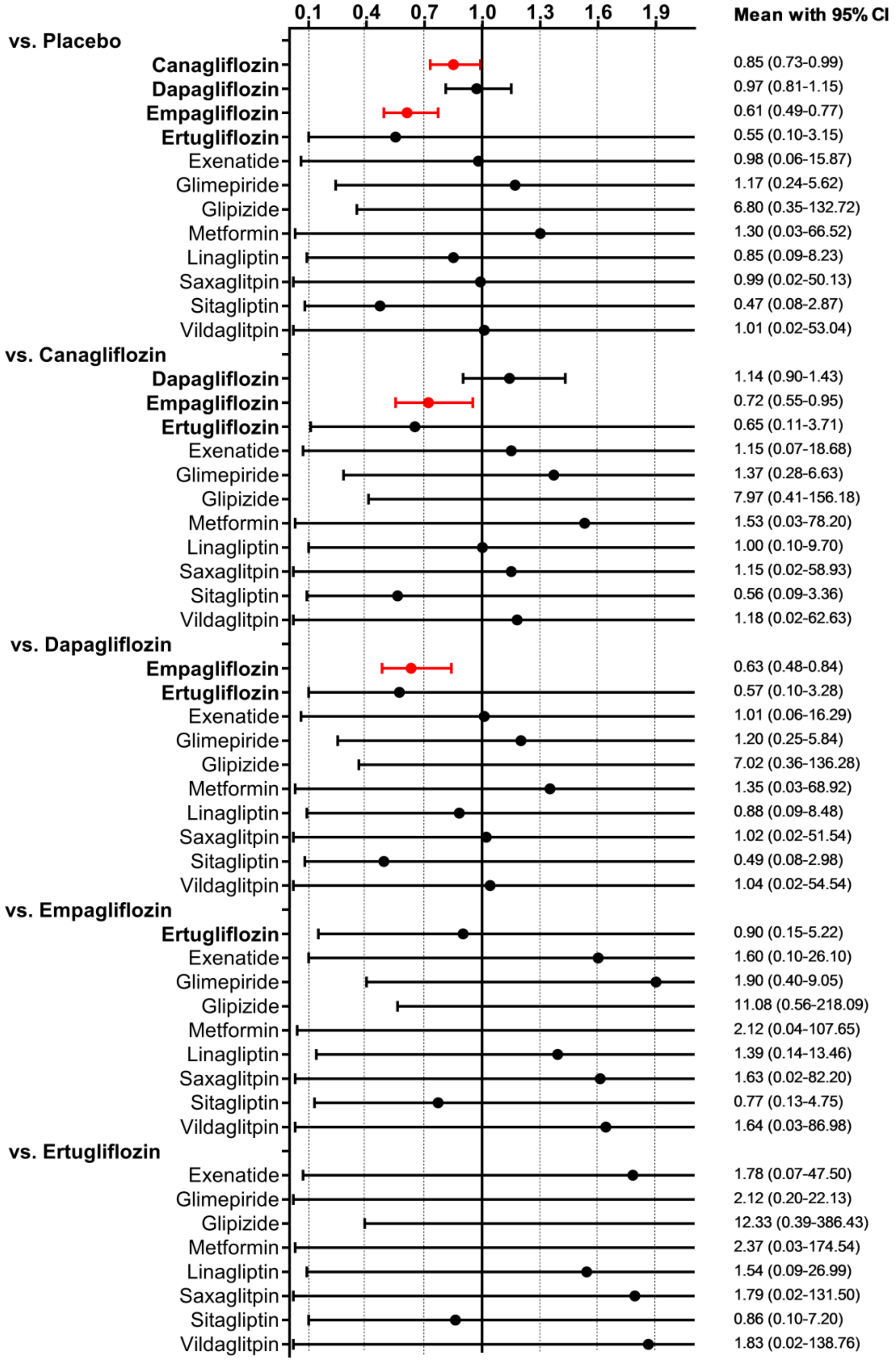

Legend: The predictive interval plot represents a forest plot of the joint estimated summary effects from both direct and indirect comparisons along with their confidence intervals. Significant estimated summary effects are shown in red colour.

eFig. 11 Predictive interval plot for worsening HF in sensitivity analyses including extension periods of trials

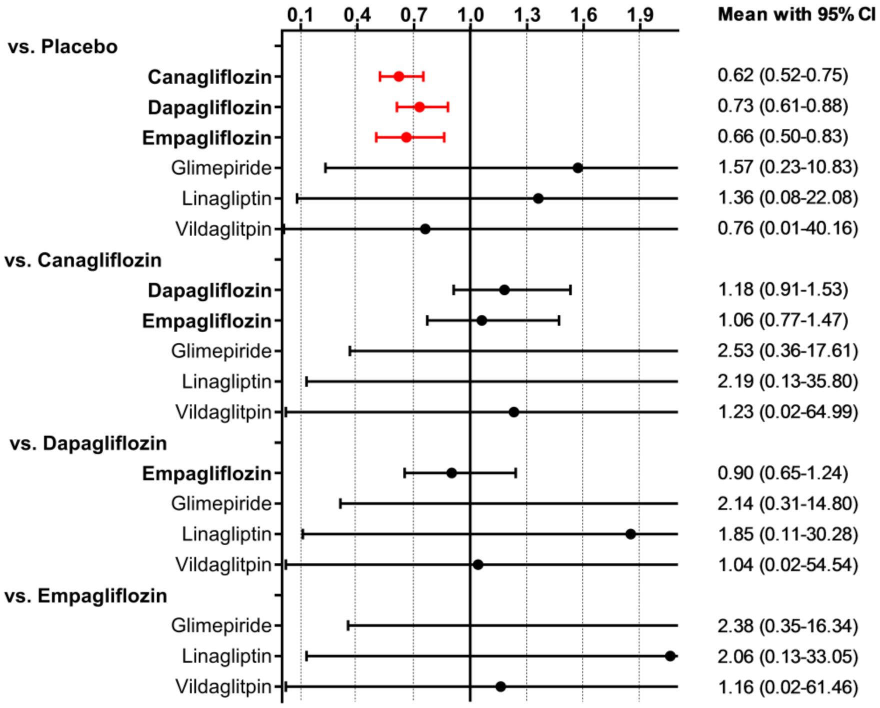

Legend: The predictive interval plot represents a forest plot of the joint estimated summary effects from both direct and indirect comparisons along with their confidence intervals. Significant estimated summary effects are shown in red colour. HF, heart failure.

eFig. 12 Predictive interval plot for all-cause mortality in sensitivity analyses excluding trials with a follow-up duration <52 weeks

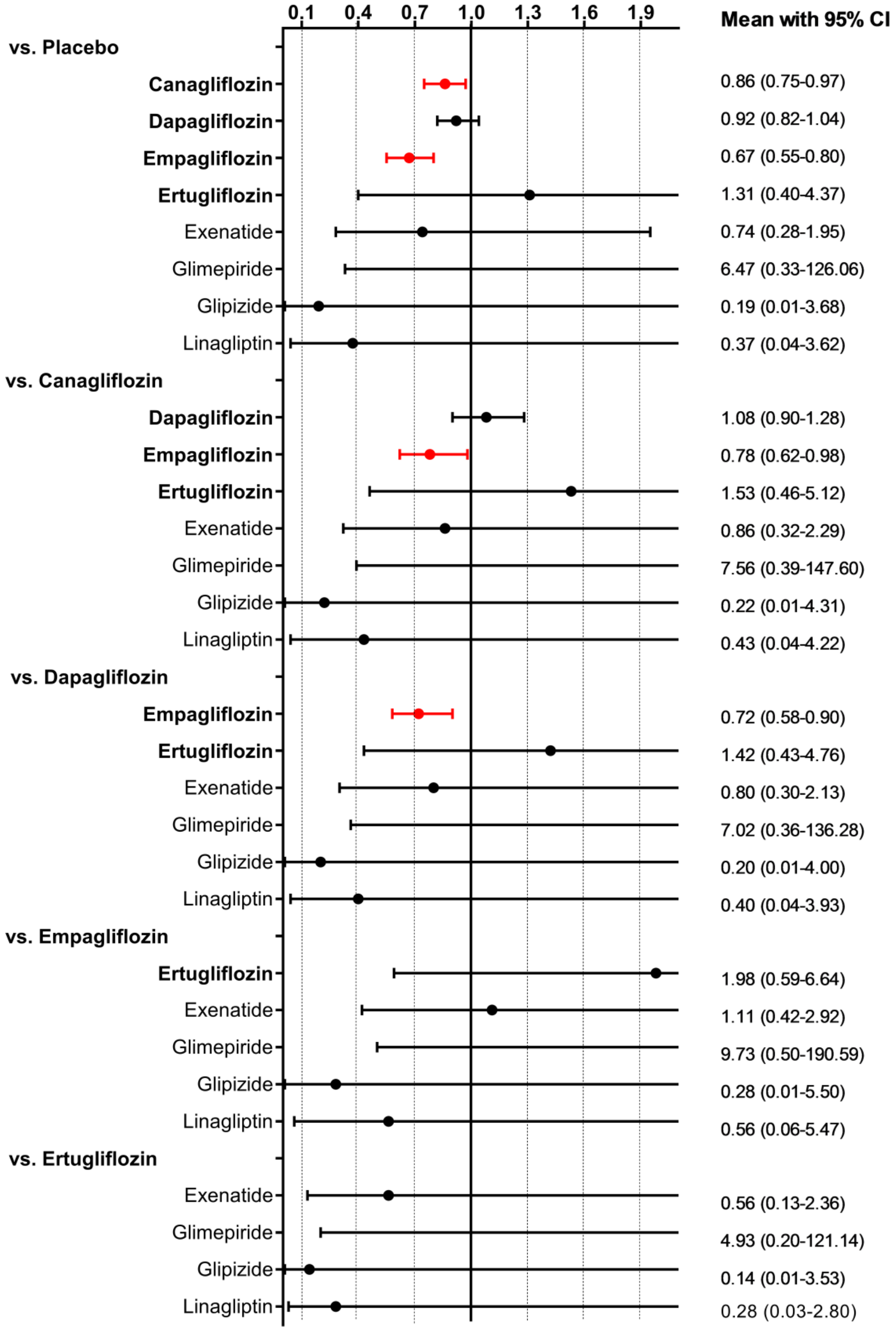

Legend: The predictive interval plot represents a forest plot of the joint estimated summary effects from both direct and indirect comparisons along with their confidence intervals.

eFig. 13 Predictive interval plot for cardiovascular mortality in sensitivity analyses excluding trials with a follow-up duration <52 weeks

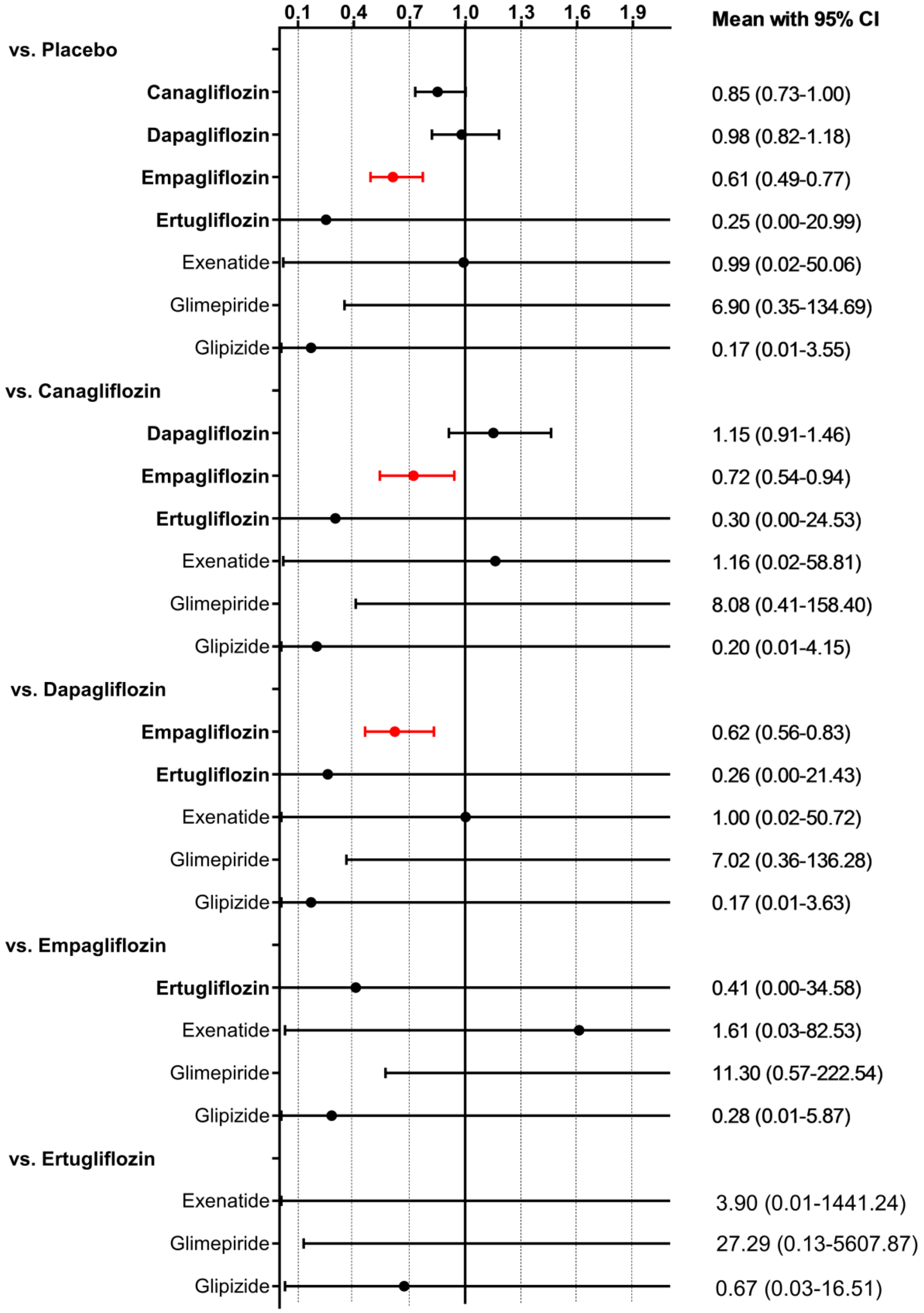

Legend: The predictive interval plot represents a forest plot of the joint estimated summary effects from both direct and indirect comparisons along with their confidence intervals.

eFig. 14 Predictive interval plot for worsening HF in sensitivity analyses excluding trials with a follow-up duration <52 weeks

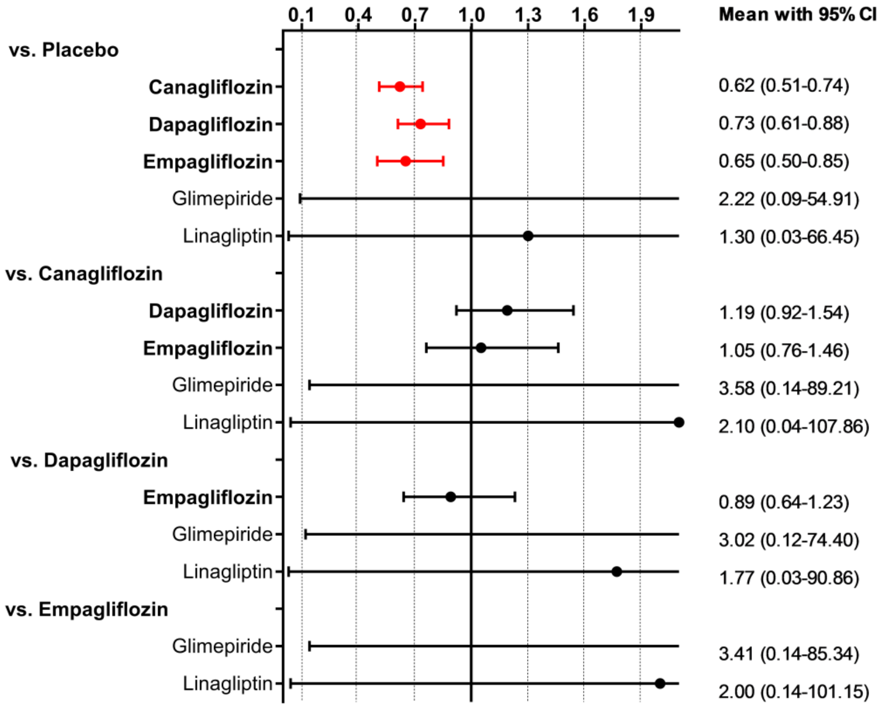

Legend: The predictive interval plot represents a forest plot of the joint estimated summary effects from both direct and indirect comparisons along with their confidence intervals. HF, heart failure.

eFig. 15 Predictive interval plot for all-cause mortality in sensitivity analyses restricted to patients included in cardiovascular outcome trials

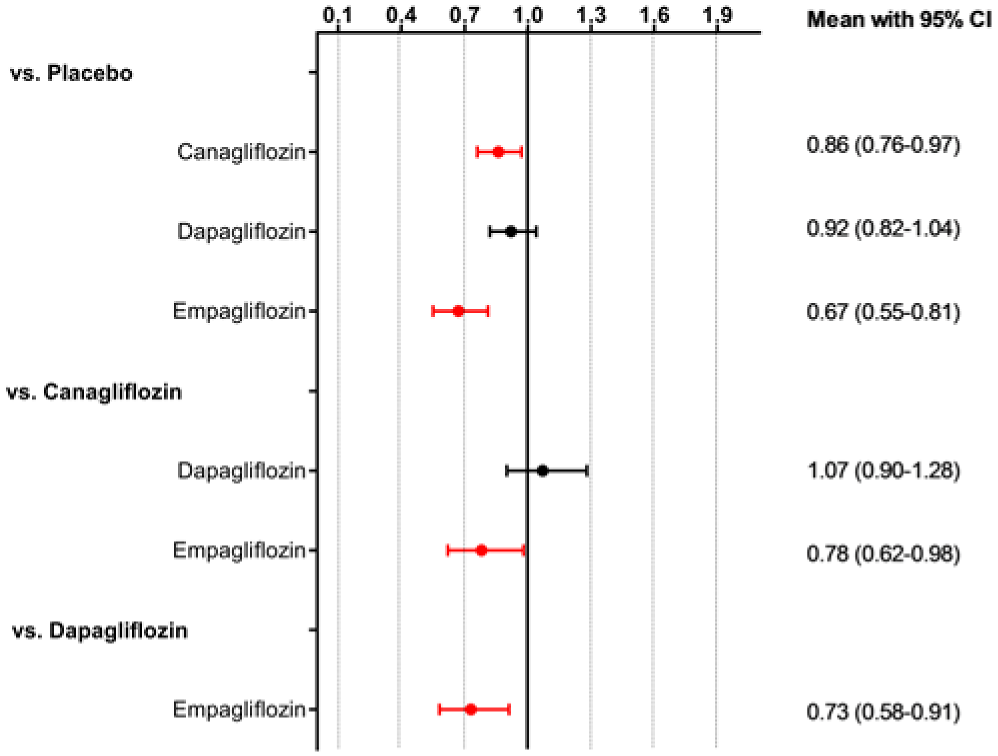

Legend: The predictive interval plot represents a forest plot of the joint estimated summary effects from both direct and indirect comparisons along with their confidence intervals.

eFig. 16 Predictive interval plot for cardiovascular mortality in sensitivity analyses restricted to patients included in cardiovascular outcome trials

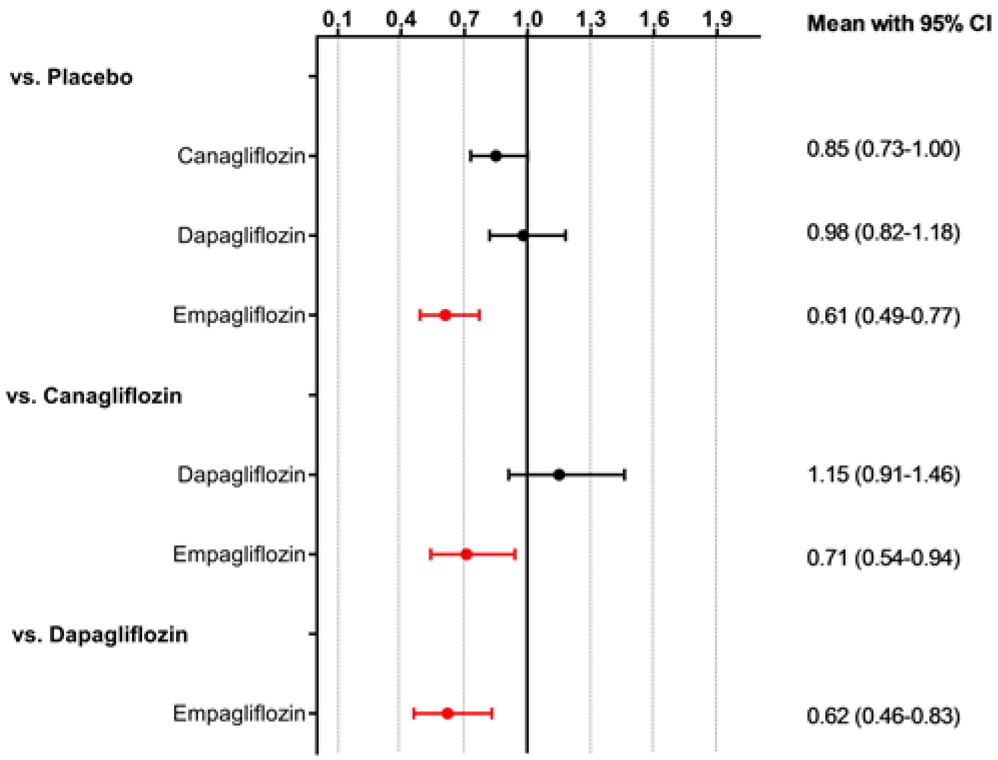

Legend: The predictive interval plot represents a forest plot of the joint estimated summary effects from both direct and indirect comparisons along with their confidence intervals.

eFig. 17 Predictive interval plot for worsening HF in sensitivity analyses restricted to patients included in cardiovascular outcome trials

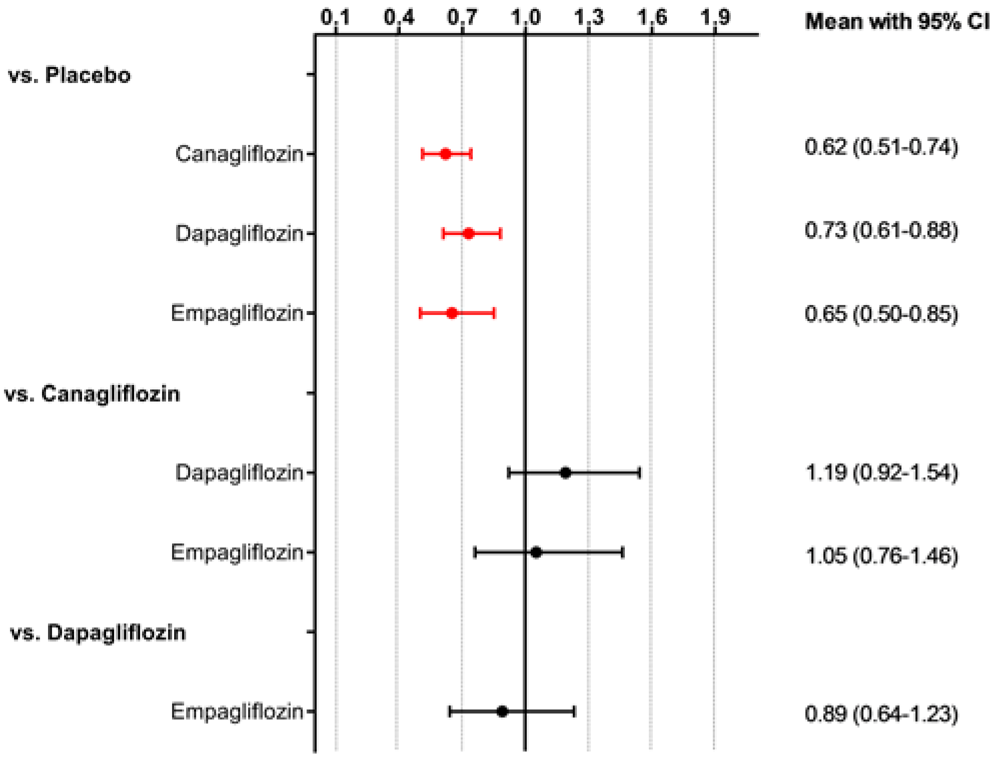

Legend: The predictive interval plot represents a forest plot of the joint estimated summary effects from both direct and indirect comparisons along with their confidence intervals. HF, heart failure.

Supplementary Tables

eTable 1 Patient characteristics

Study	Age *(years)*	Male *(n/%)*	Cardiovascular disease *(n/%)*	HbA1c *(%)*	eGFR *(ml/min/1.73m²)*	Background diabetes treatment
Bailey [1]	52.7	292 (53.5)	n.a.	7.87	n.a.	metformin
Bailey [2]	53.0	141 (50.0)	n.a.	7.9	n.a.	none
Barnett [3] [a]	63.9	430 (58.3)	n.a.	8.00	54.4	any
Bode [4]	63.6	396 (55.3)	n.a.	n.a.	n.a.	any
Bolinder [5,6]	60.7	101 (55.6)	47 (26.1)	7.2	n.a.	metformin
Cefalu [7]	56.2	756 (52.1)	n.a.	7.8	n.a.	metformin
Cefalu [8]	62.9	624 (68.3)	922 (100)	8.13	n.a.	any
Dagogo-Jack [9]	59.1	263 (56.9)	n.a.	8.0	87.9	metformin and sitagliptin
DeFronzo [10]	57.4	362 (53.7)	n.a.	7.98	89.6	metformin
Ferdinand [11]	56.8	79 (52.7)	n.a.	8.59	91.3	none or metformin only, or sulfonylurea only, or DPP-4 inhibitor only, or metformin plus sulfonylurea, or metformin plus DPP-4 inhibitor
Ferrannini [12] [a]	52.0	276 (49.5)	n.a.	8.29	n.a.	none

Fioretto [13]	66.3	182 (56.7)	n.a.	8.18	53.5	any
Forst [14]	57.4	216 (63.2)	n.a.	7.9	86.4	metformin and pioglitazone
Frias [15], Jabbour [16]	54.2	328 (47.3)	n.a.	9.3	96.9	metformin
Grunberger [17]	67.3	231 (49.5)	232 (49.7)	8.2	46.6	any except metformin, roseglitazone and SGLT2i
Hadjadj [18]	52.6	747 (54.8)	n.a.	8.46	90.4	none
Haring [19]	57.7	361 (56.7)	n.a.	7.9	89.0	metformin
Haring [20]	57.1	339 (50.7)	n.a.	8.1	87.2	metformin and sulfonylurea
Henry [21]	51.9	265 (44.3)	n.a.	9.2	n.a.	none
Henry [21]	51.6	298 (46.7)	n.a.	9.1	n.a.	none
Hollander [22,23]	58.2	642 (48.5)	n.a.	7.8	87.2	metformin
Inagaki [24]	58.0	192 (70.6)	n.a.	n.a.	n.a.	none
Jabbour [25]	55.0	248 (55.0)	n.a.	7.9	n.a.	metformin and/or sitagliptin
Ji [26]	51.3	237 (65.4)	n.a.	8.26	92.6	none
Kadowaki [27]	57.2	107 (77.5)	n.a.	8.03	84.3	tenegliptin
Kaku [28]	58.9	155 (59.4)	119 (45.6)	7.49	n.a.	none
Kohan [29]	67.0	164 (65.1)	n.a.	8.35	44.6	any
Kovacs [30,31]	54.5	241 (48.4)	n.a.	8.1	85.7	pioglitazone and/or metformin

Lavalle-Gonzalez [32]	55.4	605 (47.1)	n.a.	7.9	n.a.	metformin
Leiter [33]	63.9	644 (66.9)	965 (100)	8.05	n.a.	any
Lewin [34]	54.6	359 (53.9)	n.a.	8.02	87.5	none
Mathieu [35,36]	55.1	146 (45.6)	n.a.	8.2	92.5	metformin and sixagliptin
Matthaei [37]	61.0	118 (54.1)	186 (86.1)	8.16	n.a.	metformin and sulfonylurea
Müller-Wieland [38]	58.4	600 (63.9)	n.a.	8.3	87.1	metformin
Nauck [39]	58.5	441 (55.1)	150 (18.7)	7.7	n.a.	metformin and/or one other OAD
Neal [40]	63.3	6 509 (64.2)	6 656 (65.6)	8.2	76.5	insulin, sulfonylurea, metformin, GLP-1 receptor agonist, and/or DPP-4 inhibitor
Perkovic [41]	63.0	2 907 (66.1)	2 220 (50.4)	8.3	56.2	any
Phrommintikul [42]	63.2	26 (53.1)	49 (100)	8.2	70	any except insulin, GLP-1 receptor agonists, DPP-4 inhibitors or SGLT2i
Pollock [43]	64.4	317 (70.8)	221 (49.3)	8.5	48.9	any except GLP-1 receptor agonists, DPP-4 inhibitors or SGLT2i
Pratley [44]	55.1	664 (53.9)	n.a.	8.6	92.4	metformin
Ridderstrale [45,46]	55.9	853 (55.2)	n.a.	7.92	88.2	metformin
Rodbard [47]	57.4	121 (56.8)	n.a.	8.5	90.5	metformin and sitagliptin
Roden [48,49]	55.0	551 (61.3)	n.a.	7.88	87.4	none

Rosenstock [50]	53.5	208 (49.5)	n.a.	8.37	n.a.	pioglitazone
Rosenstock [51]	54	268 (50.2)	n.a.	8.94	94.4	metformin
Rosenstock [52]	58.8	276 (55.9)	n.a.	8.2	84	basal glargine or detemir insulin or NPH insulin, with or without metformin and/or sulphonylurea
Rosenstock [53]	54.9	569 (48.0)	n.a.	8.8	88	none
Rosenstock [54]	56.6	288 (46.4)	n.a.	8.1	90.5	metformin
Schernthaner [55]	56.7	422 (55.9)	n.a.	8.1	n.a.	metformin and sulfonylurea
Scott [56]	67.1	357 (58.1)	n.a.	7.7	n.a.	metformin and sulfonylurea
Softeland [57]	55.2	197 (60.2)	n.a.	7.97	92.3	metformin and linaglitpin
Stenlof [58]	55.4	258 (44.2)	n.a.	8.0	n.a.	none
Strojek [59,60]	58.9	285 (46.8)	212 (34.8)	7.89	n.a.	glimepiride
Terra [61]	56.4	261 (56.6)	n.a.	8.2	87.7	none
Wilding [62,63]	59.3	382 (47.8)	296 (37.0)	8.53	n.a.	insuilin and/or a maximum of two OADs
Wilding [64]	56.8	239 (51.0)	n.a.	8.1	n.a.	metformin and sulfonylurea
Wiviott [65]	63.4	10 738 (62.6)	6 974 (40.6)	8.3	85.2	any
Yale [66]	68.5	163 (60.6)	147 (54.6)	8.0	39.4	any OAD
Yang [67]	53.8	241 (54.3)	n.a.	8.13	n.a.	metformin
Yang [68]	57.5	130 (47.8)	n.a.	8.54	n.a.	≥ 20 IE insulin/day, a maximum of two OADs

Zinman [69]	63.1	5 016 (71.5)	6 964 (99.2)	8.07	74.0	any (insulin, sulfonylurea, metformin, GLP-1 receptor agonist, and/or DPP-4 inhibitor)
AstraZeneca (NCT00736879)	53.0	141 (50.0)	n.a.	7.92	n.a.	none
AstraZeneca (NCT02681094)	56.7	451 (51.1)	n.a.	n.a.	n.a.	metformin
Merck Sharp & Dohme Corp. (NCT02630706)	56.5	281 (55.5)	n.a.	8.12	99.3	metformin

[a] pooled data. DPP4, dipeptidyl peptidase-4; GLP-1, glucagon-like protein-1; NPH, Neutral Protamin Hagedorn; OAD, oral antidiabetic drug; SGLT2i, sodium-glucose cotransporter-2 inhibitor

eTable 2 Endpoint characteristics of included trials

Study	All-cause mortality		Cardiovascular mortality		Worsening HF	
	Tx (n)	Ctrl (n)	Tx (n)	Ctrl (n)	Tx (n)	Ctrl (n)
Bailey [1]	0/400	0/134	0/400	0/134	n.a.	n.a.
Bailey [2]	0/214	0/68	0/214	0/68	n.a.	n.a.
Barnett [3]	1/419	3/319	n.a.	n.a.	n.a.	n.a.
Bode [4]	3/477	0/237	n.a.	n.a.	1/477	0/237
Bolinder [5,6]	1/91<br>[1/91]	0/91<br>[0/91]	0/91<br>[0/91]	0/91<br>[0/91]	n.a.<br>[n.a.]	n.a.<br>[n.a.]
Cefalu [7]	2/968	2/482	n.a.	n.a.	n.a.	n.a.
Cefalu [8]	2/455<br>[7/455]	1/459<br>[2/459]	n.a.<br>[n.a.]	n.a.<br>[n.a.]	n.a.<br>[n.a.]	n.a.<br>[n.a.]
Dagogo-Jack [9]	0/309<br>[0/309]	0/153<br>[0/153]	0/309<br>[0/309]	0/153<br>[0/153]	n.a.<br>[n.a.]	n.a.<br>[n.a.]
DeFronzo [10]	n.a.<br>[1/281]	n.a.<br>[0/132]	n.a.<br>[0/281]	n.a.<br>[0/132]	n.a.<br>[0/281]	n.a.<br>[0/132]
Ferdinand [11]	0/82	0/80	0/82	0/80	1/82	0/80
Ferrannini [12]	1/483	0/75	0/483	0/75	n.a.	n.a.
Fioretto [13]	n.a.	n.a.	0/160	0/161	n.a.	n.a.

Forst [14]	0/227	0/115	0/227	0/115	n.a.	n.a.
Frias [15], Jabbour [16]	1/233<br>[1/233]	1/230<br>[1/230]	1/233<br>[1/233]	1/230<br>[1/230]	n.a.<br>[n.a.]	n.a.<br>[n.a.]
Grunberger [17]	7/313	3/154	n.a.	n.a.	n.a.	n.a.
Hadjadj [18]	0/333	0/332	0/333	0/332	n.a.	n.a.
Haring [19]	1/441	0/225	1/441	0/225	n.a.	n.a.
Haring [20]	0/430	0/207	0/430	0/207	n.a.	n.a.
Henry [21]	1/203	0/201	1/203	0/201	n.a.	n.a.
Henry [21]	0/219	1/208	0/219	1/208	n.a.	n.a.
Hollander [22,23]	6/888<br>[9/880]	0/437<br>[1/435]	n.a.<br>[n.a.]	n.a.<br>[n.a.]	n.a.<br>[n.a.]	n.a.<br>[n.a.]
Inagaki [24]	0/179	0/93	0/179	0/93	n.a.	n.a.
Jabbour [25]	0/225<br>[0/225]	1/226<br>[1/226]	0/225<br>[0/225]	0/226<br>[0/226]	n.a.<br>[n.a.]	n.a.<br>[n.a.]
Ji [26]	0/261	0/132	0/261	0/132	n.a.	n.a.
Kadowaki [27]	0/70	0/68	0/70	0/68	n.a.	n.a.
Kaku [28]	0/174	0/87	0/174	0/87	n.a.	n.a.
Kohan [29]	1/168<br>[5/168]	2/84<br>[5/84]	n.a.<br>[4/168]	n.a.<br>[3/168]	n.a.<br>[n.a.]	n.a.<br>[n.a.]

Kovacs [30,31]	2/333<br>[4/233]	1/165<br>[1/165]	n.a.<br>[3/233]	n.a.<br>[1/165]	n.a.<br>[n.a.]	n.a.<br>[n.a.]
Lavalle-Gonzalez [32]	1/735	1/366 and 1/183 [a]	n.a.	n.a.	n.a.	n.a.
Leiter [33]	2/482<br>[5/482]	1/483<br>[4/483]	n.a.<br>[n.a.]	n.a.<br>[n.a.]	n.a.<br>[n.a.]	n.a.<br>[n.a.]
Lewin [34]	3/270	0/135	n.a.	n.a.	0/270	0/135
Mathieu [35,36]	0/160<br>[1/160]	0/160<br>[0/160]	0/160<br>[n.a.]	0/160<br>[n.a.]	2/160<br>[3/160]	0/160<br>[2/160]
Matthaei [37]	0/108	0/108	0/108	0/108	n.a.	n.a.
Müller-Wieland [38]	0/314	0/313	0/314	0/313	0/314	1/313
Nauck [39]	0/406	3/408	0/406	3/408	n.a.	n.a.
Neal [40]	362/5 795	306/4 347	243/5 795	201/4 347	115/5 795	137/4 347
Perkovic [41]	168/2 202	201/2 199	110/2 202	140/2 199	89/2 202	141/2 199
Phrommintikul [42]	0/25	0/24	0/25	0/24	n.a.	n.a.
Pollock [43]	1/144	0/148	n.a.	n.a.	1/144	0/148
Pratley [44]	1/498	0/247	1/498	0/247	n.a.	n.a.
Ridderstrale [45,46]	5/765<br>[8/765]	5/780<br>[8/780]	n.a.<br>[2/765]	n.a.<br>[4/780]	n.a.<br>[1/765]	n.a.<br>[2/780]
Rodbard [47]	0/108	0/108	0/108	0/108	n.a.	n.a.

Roden [48,49]	0/448<br>[0/448]	0/223 and 1/228 [a]<br>[1/223 and 1/228] [a]	n.a.<br>[n.a.]	n.a.<br>[n.a.]	n.a.<br>[n.a.]	n.a.<br>[n.a.]
Rosenstock [50]	1/281	0/139	0/281	0/139	0/281	1/139
Rosenstock [51]	0/179	0/176	0/179	0/176	n.a.	n.a.
Rosenstock [52]	0/324	1/170	n.a.	n.a.	n.a.	n.a.
Rosenstock [53]	0/475	1/237	n.a.	n.a.	n.a.	n.a.
Rosenstock [54]	0/412	0/209	0/412	0/209	n.a.	n.a.
Schernthaner [55]	2/377	0/378	2/377	0/378	n.a.	n.a.
Scott [56]	0/306	0/307	0/306	0/307	n.a.	n.a.
Softeland [57]	0/222	0/110	0/222	0/110	n.a.	n.a.
Stenlof [58]	1/392	1/192	0/392	0/192	n.a.	n.a.
Strojek [59,60]	2/450<br>[3/450]	0/146<br>[0/146]	2/450<br>[3/450]	0/146<br>[0/146]	n.a.<br>[n.a.]	n.a.<br>[n.a.]
Terra [61]	0/305	0/156	0/305	0/156	n.a.	n.a.
Wilding [62,63]	1/607<br>[3/610]	0/197<br>[0/197]	n.a.<br>[3/610]	n.a.<br>[0/197]	n.a.<br>[n.a.]	n.a.<br>[n.a.]
Wilding [64]	0/313	0/156	0/313	0/156	n.a.	n.a.
Wiviott [65]	529/8 582	570/8 578	245/8 582	249/ 8578	212/8 582	286/ 8578
Yale [66]	1/179	1/90	n.a.	n.a.	n.a.	n.a.

Yang [67]	0/299	0/145	0/299	0/145	n.a.	n.a.
Yang [68]	0/139	0/133	0/139	0/133	n.a.	n.a.
Zinman [69]	269/4 687	194/2 333	172/4 687	137/2 333	126/4 687	95/2 333
AstraZeneca (NCT00736879)	0/214	0/68	0/214	0/68	n.a.	n.a.
AstraZeneca (NCT02681094)	2/294	0/296	n.a.	n.a.	n.a.	n.a.
Merck Sharp & Dohme Corp. (NCT02630706)	0/339	0/167	0/339	0/167	n.a.	n.a.

Ctrl, control; HF, heart failure; n.a., not available; Tx, treatment. [a] This study comprised two comparators. Numbers in brackets represent results of the extension periods of the respective trials.

Supplementary References

1. Bailey CJ, Gross JL, Pieters A, Bastien A, List JF (2010) Effect of dapagliflozin in patients with type 2 diabetes who have inadequate glycaemic control with metformin: a randomised, double-blind, placebo-controlled trial. Lancet 375 (9733):2223-2233. doi:10.1016/S0140-6736(10)60407-2

2. Bailey CJ, Iqbal N, T'Joen C, List JF (2012) Dapagliflozin monotherapy in drug-naive patients with diabetes: a randomized-controlled trial of low-dose range. Diabetes, obesity & metabolism 14 (10):951-959. doi:10.1111/j.1463-1326.2012.01659.x

3. Barnett AH, Mithal A, Manassie J, Jones R, Rattunde H, Woerle HJ, Broedl UC, investigators E-RRt (2014) Efficacy and safety of empagliflozin added to existing antidiabetes treatment in patients with type 2 diabetes and chronic kidney disease: a randomised, double-blind, placebo-controlled trial. The lancet Diabetes & endocrinology 2 (5):369-384. doi:10.1016/S2213-8587(13)70208-0

4. Bode B, Stenlof K, Sullivan D, Fung A, Usiskin K (2013) Efficacy and safety of canagliflozin treatment in older subjects with type 2 diabetes mellitus: a randomized trial. Hosp Pract (1995) 41 (2):72-84. doi:10.3810/hp.2013.04.1020

5. Bolinder J, Ljunggren O, Kullberg J, Johansson L, Wilding J, Langkilde AM, Sugg J, Parikh S (2012) Effects of dapagliflozin on body weight, total fat mass, and regional adipose tissue distribution in patients with type 2 diabetes mellitus with inadequate glycemic control on metformin. J Clin Endocrinol Metab 97 (3):1020-1031. doi:10.1210/jc.2011-2260

6. Bolinder J, Ljunggren O, Johansson L, Wilding J, Langkilde AM, Sjostrom CD, Sugg J, Parikh S (2014) Dapagliflozin maintains glycaemic control while reducing weight and body fat mass over 2 years in patients with type 2 diabetes mellitus inadequately controlled on metformin. Diabetes, obesity & metabolism 16 (2):159-169. doi:10.1111/dom.12189

7. Cefalu WT, Leiter LA, Yoon KH, Arias P, Niskanen L, Xie J, Balis DA, Canovatchel W, Meininger G (2013) Efficacy and safety of canagliflozin versus glimepiride in patients with type 2 diabetes inadequately controlled with metformin (CANTATA-SU): 52 week results from a randomised,

double-blind, phase 3 non-inferiority trial. Lancet 382 (9896):941-950. doi:10.1016/S0140-6736(13)60683-2

8. Cefalu WT, Leiter LA, de Bruin TW, Gause-Nilsson I, Sugg J, Parikh SJ (2015) Dapagliflozin's Effects on Glycemia and Cardiovascular Risk Factors in High-Risk Patients With Type 2 Diabetes: A 24-Week, Multicenter, Randomized, Double-Blind, Placebo-Controlled Study With a 28-Week Extension. Diabetes care 38 (7):1218-1227. doi:10.2337/dc14-0315

9. Dagogo-Jack S, Liu J, Eldor R, Amorin G, Johnson J, Hille D, Liao Y, Huyck S, Golm G, Terra SG, Mancuso JP, Engel SS, Lauring B (2018) Efficacy and safety of the addition of ertugliflozin in patients with type 2 diabetes mellitus inadequately controlled with metformin and sitagliptin: The VERTIS SITA2 placebo-controlled randomized study. Diabetes, obesity & metabolism 20 (3):530-540. doi:10.1111/dom.13116

10. DeFronzo RA, Lewin A, Patel S, Liu D, Kaste R, Woerle HJ, Broedl UC (2015) Combination of empagliflozin and linagliptin as second-line therapy in subjects with type 2 diabetes inadequately controlled on metformin. Diabetes care 38 (3):384-393. doi:10.2337/dc14-2364

11. Ferdinand KC, Izzo JL, Lee J, Meng L, George J, Salsali A, Seman L (2019) Antihyperglycemic and Blood Pressure Effects of Empagliflozin in African Americans with Type 2 Diabetes and Hypertension. Circulation. doi:10.1161/CIRCULATIONAHA.118.036568

12. Ferrannini E, Ramos SJ, Salsali A, Tang W, List JF (2010) Dapagliflozin monotherapy in type 2 diabetic patients with inadequate glycemic control by diet and exercise: a randomized, double-blind, placebo-controlled, phase 3 trial. Diabetes care 33 (10):2217-2224. doi:10.2337/dc10-0612

13. Fioretto P, Del Prato S, Buse JB, Goldenberg R, Giorgino F, Reyner D, Langkilde AM, Sjostrom CD, Sartipy P, Investigators DS (2018) Efficacy and safety of dapagliflozin in patients with type 2 diabetes and moderate renal impairment (chronic kidney disease stage 3A): The DERIVE Study. Diabetes, obesity & metabolism 20 (11):2532-2540. doi:10.1111/dom.13413

14. Forst T, Guthrie R, Goldenberg R, Yee J, Vijapurkar U, Meininger G, Stein P (2014) Efficacy and safety of canagliflozin over 52 weeks in patients with type 2 diabetes on background metformin and pioglitazone. Diabetes, obesity & metabolism 16 (5):467-477. doi:10.1111/dom.12273

15. Frias JP, Guja C, Hardy E, Ahmed A, Dong F, Ohman P, Jabbour SA (2016) Exenatide once weekly plus dapagliflozin once daily versus exenatide or dapagliflozin alone in patients with type 2 diabetes inadequately controlled with metformin monotherapy (DURATION-8): a 28 week, multicentre, double-blind, phase 3, randomised controlled trial. The lancet Diabetes & endocrinology 4 (12):1004-1016. doi:10.1016/S2213-8587(16)30267-4

16. Jabbour SA, Frias JP, Hardy E, Ahmed A, Wang H, Ohman P, Guja C (2018) Safety and Efficacy of Exenatide Once Weekly Plus Dapagliflozin Once Daily Versus Exenatide or Dapagliflozin Alone in Patients With Type 2 Diabetes Inadequately Controlled With Metformin Monotherapy: 52-Week Results of the DURATION-8 Randomized Controlled Trial. Diabetes care 41 (10):2136-2146. doi:10.2337/dc18-0680

17. Grunberger G, Camp S, Johnson J, Huyck S, Terra SG, Mancuso JP, Jiang ZW, Golm G, Engel SS, Lauring B (2018) Ertugliflozin in Patients with Stage 3 Chronic Kidney Disease and Type 2 Diabetes Mellitus: The VERTIS RENAL Randomized Study. Diabetes therapy : research, treatment and education of diabetes and related disorders 9 (1):49-66. doi:10.1007/s13300-017-0337-5

18. Hadjadj S, Rosenstock J, Meinicke T, Woerle HJ, Broedl UC (2016) Initial Combination of Empagliflozin and Metformin in Patients With Type 2 Diabetes. Diabetes care 39 (10):1718-1728. doi:10.2337/dc16-0522

19. Haring HU, Merker L, Seewaldt-Becker E, Weimer M, Meinicke T, Woerle HJ, Broedl UC, Investigators E-RMT (2013) Empagliflozin as add-on to metformin plus sulfonylurea in patients with type 2 diabetes: a 24-week, randomized, double-blind, placebo-controlled trial. Diabetes care 36 (11):3396-3404. doi:10.2337/dc12-2673

20. Haring HU, Merker L, Seewaldt-Becker E, Weimer M, Meinicke T, Broedl UC, Woerle HJ, Investigators E-RMT (2014) Empagliflozin as add-on to metformin in patients with type 2 diabetes: a 24-week, randomized, double-blind, placebo-controlled trial. Diabetes care 37 (6):1650-1659. doi:10.2337/dc13-2105

21. Henry RR, Murray AV, Marmolejo MH, Hennicken D, Ptaszynska A, List JF (2012) Dapagliflozin, metformin XR, or both: initial pharmacotherapy for type 2 diabetes, a randomised controlled trial. Int J Clin Pract 66 (5):446-456. doi:10.1111/j.1742-1241.2012.02911.x

22. Hollander P, Liu J, Hill J, Johnson J, Jiang ZW, Golm G, Huyck S, Terra SG, Mancuso JP, Engel SS, Lauring B (2018) Ertugliflozin Compared with Glimepiride in Patients with Type 2 Diabetes Mellitus Inadequately Controlled on Metformin: The VERTIS SU Randomized Study. Diabetes therapy : research, treatment and education of diabetes and related disorders 9 (1):193-207. doi:10.1007/s13300-017-0354-4

23. Hollander P, Liu J, Hill J, Johnson J, Jiang ZW, Wang D, Golm G, Huyck S, Terra S, Mancuso JP, et al. (2018) Safety and efficacy of ertugliflozin compared with glimepiride after 104 weeks in patients with type 2 diabetes inadequately controlled on metformin: VERTIS SU extension. Diabetologia Conference: 54th annual meeting of the european association for the study diabetes, EASD 2018 Germany 61 (Supplement 1):S304-S305. doi:10.1007/s00125-018-4693-0

24. Inagaki N, Kondo K, Yoshinari T, Takahashi N, Susuta Y, Kuki H (2014) Efficacy and safety of canagliflozin monotherapy in Japanese patients with type 2 diabetes inadequately controlled with diet and exercise: a 24-week, randomized, double-blind, placebo-controlled, Phase III study. Expert opinion on pharmacotherapy 15 (11):1501-1515. doi:10.1517/14656566.2014.935764

25. Jabbour SA, Hardy E, Sugg J, Parikh S, Study G (2014) Dapagliflozin is effective as add-on therapy to sitagliptin with or without metformin: a 24-week, multicenter, randomized, double-blind, placebo-controlled study. Diabetes care 37 (3):740-750. doi:10.2337/dc13-0467

26. Ji L, Ma J, Li H, Mansfield TA, T'Joen CL, Iqbal N, Ptaszynska A, List JF (2014) Dapagliflozin as monotherapy in drug-naive Asian patients with type 2 diabetes mellitus: a randomized, blinded, prospective phase III study. Clinical therapeutics 36 (1):84-100.e109. doi:10.1016/j.clinthera.2013.11.002

27. Kadowaki T, Inagaki N, Kondo K, Nishimura K, Kaneko G, Maruyama N, Nakanishi N, Iijima H, Watanabe Y, Gouda M (2017) Efficacy and safety of canagliflozin as add-on therapy to teneligliptin in Japanese patients with type 2 diabetes mellitus: Results of a 24-week, randomized, double-blind, placebo-controlled trial. Diabetes, obesity & metabolism 19 (6):874-882. doi:10.1111/dom.12898

28. Kaku K, Kiyosue A, Inoue S, Ueda N, Tokudome T, Yang J, Langkilde AM (2014) Efficacy and safety of dapagliflozin monotherapy in Japanese patients with type 2 diabetes inadequately controlled by diet and exercise. Diabetes, obesity & metabolism 16 (11):1102-1110. doi:10.1111/dom.12325

29. Kohan DE, Fioretto P, Tang W, List JF (2014) Long-term study of patients with type 2 diabetes and moderate renal impairment shows that dapagliflozin reduces weight and blood pressure but does not improve glycemic control. Kidney Int 85 (4):962-971. doi:10.1038/ki.2013.356

30. Kovacs CS, Seshiah V, Swallow R, Jones R, Rattunde H, Woerle HJ, Broedl UC, investigators E-RPt (2014) Empagliflozin improves glycaemic and weight control as add-on therapy to pioglitazone or pioglitazone plus metformin in patients with type 2 diabetes: a 24-week, randomized, placebo-controlled trial. Diabetes, obesity & metabolism 16 (2):147-158. doi:10.1111/dom.12188

31. Kovacs CS, Seshiah V, Merker L, Christiansen AV, Roux F, Salsali A, Kim G, Stella P, Woerle HJ, Broedl UC, investigators E-REP (2015) Empagliflozin as Add-on Therapy to Pioglitazone With or Without Metformin in Patients With Type 2 Diabetes Mellitus. Clinical therapeutics 37 (8):1773-1788 e1771. doi:10.1016/j.clinthera.2015.05.511

32. Lavalle-Gonzalez FJ, Januszewicz A, Davidson J, Tong C, Qiu R, Canovatchel W, Meininger G (2013) Efficacy and safety of canagliflozin compared with placebo and sitagliptin in patients with type 2 diabetes on background metformin monotherapy: a randomised trial. Diabetologia 56 (12):2582-2592. doi:10.1007/s00125-013-3039-1

33. Leiter LA, Cefalu WT, de Bruin TW, Gause-Nilsson I, Sugg J, Parikh SJ (2014) Dapagliflozin added to usual care in individuals with type 2 diabetes mellitus with preexisting cardiovascular disease: a 24-week, multicenter, randomized, double-blind, placebo-controlled study with a 28-week extension. J Am Geriatr Soc 62 (7):1252-1262. doi:10.1111/jgs.12881

34. Lewin A, DeFronzo RA, Patel S, Liu D, Kaste R, Woerle HJ, Broedl UC (2015) Initial combination of empagliflozin and linagliptin in subjects with type 2 diabetes. Diabetes care 38 (3):394-402. doi:10.2337/dc14-2365

35. Mathieu C, Herrera Marmolejo M, Gonzalez Gonzalez JG, Hansen L, Chen H, Johnsson E, Garcia-Sanchez R, Iqbal N (2016) Efficacy and safety of triple therapy with dapagliflozin add-on to saxagliptin plus metformin over 52 weeks in patients with type 2 diabetes. Diabetes, obesity & metabolism 18 (11):1134-1137. doi:10.1111/dom.12737

36. Mathieu C, Ranetti AE, Li D, Ekholm E, Cook W, Hirshberg B, Chen H, Hansen L, Iqbal N (2015) Randomized, Double-Blind, Phase 3 Trial of Triple Therapy With Dapagliflozin Add-on to

Saxagliptin Plus Metformin in Type 2 Diabetes. Diabetes care 38 (11):2009-2017. doi:10.2337/dc15-0779

37. Matthaei S, Bowering K, Rohwedder K, Grohl A, Parikh S, Study G (2015) Dapagliflozin improves glycemic control and reduces body weight as add-on therapy to metformin plus sulfonylurea: a 24-week randomized, double-blind clinical trial. Diabetes care 38 (3):365-372. doi:10.2337/dc14-0666

38. Muller-Wieland D, Kellerer M, Cypryk K, Skripova D, Rohwedder K, Johnsson E, Garcia-Sanchez R, Kurlyandskaya R, Sjostrom CD, Jacob S, Seufert J, Dronamraju N, Csomos K (2018) Efficacy and safety of dapagliflozin or dapagliflozin plus saxagliptin versus glimepiride as add-on to metformin in patients with type 2 diabetes. Diabetes, obesity & metabolism 20 (11):2598-2607. doi:10.1111/dom.13437

39. Nauck MA, Del Prato S, Meier JJ, Duran-Garcia S, Rohwedder K, Elze M, Parikh SJ (2011) Dapagliflozin versus glipizide as add-on therapy in patients with type 2 diabetes who have inadequate glycemic control with metformin: a randomized, 52-week, double-blind, active-controlled noninferiority trial. Diabetes care 34 (9):2015-2022. doi:10.2337/dc11-0606

40. Neal B, Perkovic V, Mahaffey KW, de Zeeuw D, Fulcher G, Erondu N, Shaw W, Law G, Desai M, Matthews DR (2017) Canagliflozin and Cardiovascular and Renal Events in Type 2 Diabetes. The New England journal of medicine 377 (7):644-657. doi:10.1056/NEJMoa1611925

41. Perkovic V, Jardine MJ, Neal B, Bompoint S, Heerspink HJL, Charytan DM, Edwards R, Agarwal R, Bakris G, Bull S, Cannon CP, Capuano G, Chu PL, de Zeeuw D, Greene T, Levin A, Pollock C, Wheeler DC, Yavin Y, Zhang H, Zinman B, Meininger G, Brenner BM, Mahaffey KW (2019) Canagliflozin and Renal Outcomes in Type 2 Diabetes and Nephropathy. N Engl J Med 380 (24):2295-2306. doi:10.1056/NEJMoa1811744

42. Phrommintikul A, Wongcharoen W, Kumfu S, Jaiwongkam T, Gunaparn S, Chattipakorn S, Chattipakorn N (2019) Effects of dapagliflozin vs vildagliptin on cardiometabolic parameters in diabetic patients with coronary artery disease: a randomised study. British journal of clinical pharmacology 85 (6):1337-1347. doi:10.1111/bcp.13903

43. Pollock C, Stefansson B, Reyner D, Rossing P, Sjostrom CD, Wheeler DC, Langkilde AM, Heerspink HJL (2019) Albuminuria-lowering effect of dapagliflozin alone and in combination with saxagliptin and effect of dapagliflozin and saxagliptin on glycaemic control in patients with type 2 diabetes and chronic kidney disease (DELIGHT): a randomised, double-blind, placebo-controlled trial. The lancet Diabetes & endocrinology 7 (6):429-441. doi:10.1016/S2213-8587(19)30086-5
44. Pratley RE, Eldor R, Raji A, Golm G, Huyck SB, Qiu Y, Sunga S, Johnson J, Terra SG, Mancuso JP, Engel SS, Lauring B (2018) Ertugliflozin plus sitagliptin versus either individual agent over 52 weeks in patients with type 2 diabetes mellitus inadequately controlled with metformin: The VERTIS FACTORIAL randomized trial. Diabetes, obesity & metabolism 20 (5):1111-1120. doi:10.1111/dom.13194
45. Ridderstrale M, Andersen KR, Zeller C, Kim G, Woerle HJ, Broedl UC, investigators E-RHHSt (2014) Comparison of empagliflozin and glimepiride as add-on to metformin in patients with type 2 diabetes: a 104-week randomised, active-controlled, double-blind, phase 3 trial. The lancet Diabetes & endocrinology 2 (9):691-700. doi:10.1016/S2213-8587(14)70120-2
46. Ridderstrale M, Rosenstock J, Andersen KR, Woerle HJ, Salsali A, investigators E-RHHSt (2018) Empagliflozin compared with glimepiride in metformin-treated patients with type 2 diabetes: 208-week data from a masked randomized controlled trial. Diabetes, obesity & metabolism 20 (12):2768-2777. doi:10.1111/dom.13457
47. Rodbard HW, Seufert J, Aggarwal N, Cao A, Fung A, Pfeifer M, Alba M (2016) Efficacy and safety of titrated canagliflozin in patients with type 2 diabetes mellitus inadequately controlled on metformin and sitagliptin. Diabetes, obesity & metabolism 18 (8):812-819. doi:10.1111/dom.12684
48. Roden M, Merker L, Christiansen AV, Roux F, Salsali A, Kim G, Stella P, Woerle HJ, Broedl UC, investigators E-REM (2015) Safety, tolerability and effects on cardiometabolic risk factors of empagliflozin monotherapy in drug-naive patients with type 2 diabetes: a double-blind extension of a Phase III randomized controlled trial. Cardiovascular diabetology 14:154. doi:10.1186/s12933-015-0314-0
49. Roden M, Weng J, Eilbracht J, Delafont B, Kim G, Woerle HJ, Broedl UC, investigators E-RMt (2013) Empagliflozin monotherapy with sitagliptin as an active comparator in patients with type 2

diabetes: a randomised, double-blind, placebo-controlled, phase 3 trial. The lancet Diabetes & endocrinology 1 (3):208-219. doi:10.1016/S2213-8587(13)70084-6

50. Rosenstock J, Vico M, Wei L, Salsali A, List JF (2012) Effects of dapagliflozin, an SGLT2 inhibitor, on HbA(1c), body weight, and hypoglycemia risk in patients with type 2 diabetes inadequately controlled on pioglitazone monotherapy. Diabetes Care 35 (7):1473-1478. doi:10.2337/dc11-1693

51. Rosenstock J, Hansen L, Zee P, Li Y, Cook W, Hirshberg B, Iqbal N (2015) Dual add-on therapy in type 2 diabetes poorly controlled with metformin monotherapy: a randomized double-blind trial of saxagliptin plus dapagliflozin addition versus single addition of saxagliptin or dapagliflozin to metformin. Diabetes care 38 (3):376-383. doi:10.2337/dc14-1142

52. Rosenstock J, Jelaska A, Zeller C, Kim G, Broedl UC, Woerle HJ, investigators E-RBt (2015) Impact of empagliflozin added on to basal insulin in type 2 diabetes inadequately controlled on basal insulin: a 78-week randomized, double-blind, placebo-controlled trial. Diabetes, obesity & metabolism 17 (10):936-948. doi:10.1111/dom.12503

53. Rosenstock J, Chuck L, Gonzalez-Ortiz M, Merton K, Craig J, Capuano G, Qiu R (2016) Initial Combination Therapy With Canagliflozin Plus Metformin Versus Each Component as Monotherapy for Drug-Naive Type 2 Diabetes. Diabetes care 39 (3):353-362. doi:10.2337/dc15-1736

54. Rosenstock J, Frias J, Pall D, Charbonnel B, Pascu R, Saur D, Darekar A, Huyck S, Shi H, Lauring B, Terra SG (2018) Effect of ertugliflozin on glucose control, body weight, blood pressure and bone density in type 2 diabetes mellitus inadequately controlled on metformin monotherapy (VERTIS MET). Diabetes, obesity & metabolism 20 (3):520-529. doi:10.1111/dom.13103

55. Schernthaner G, Gross JL, Rosenstock J, Guarisco M, Fu M, Yee J, Kawaguchi M, Canovatchel W, Meininger G (2013) Canagliflozin compared with sitagliptin for patients with type 2 diabetes who do not have adequate glycemic control with metformin plus sulfonylurea: a 52-week randomized trial. Diabetes care 36 (9):2508-2515. doi:10.2337/dc12-2491

56. Scott R, Morgan J, Zimmer Z, Lam RLH, O'Neill EA, Kaufman KD, Engel SS, Raji A (2018) A randomized clinical trial of the efficacy and safety of sitagliptin compared with dapagliflozin in

patients with type 2 diabetes mellitus and mild renal insufficiency: The CompoSIT-R study. Diabetes, obesity & metabolism 20 (12):2876-2884. doi:10.1111/dom.13473

57. Softeland E, Meier JJ, Vangen B, Toorawa R, Maldonado-Lutomirsky M, Broedl UC (2017) Empagliflozin as Add-on Therapy in Patients With Type 2 Diabetes Inadequately Controlled With Linagliptin and Metformin: A 24-Week Randomized, Double-Blind, Parallel-Group Trial. Diabetes care 40 (2):201-209. doi:10.2337/dc16-1347

58. Stenlof K, Cefalu WT, Kim KA, Alba M, Usiskin K, Tong C, Canovatchel W, Meininger G (2013) Efficacy and safety of canagliflozin monotherapy in subjects with type 2 diabetes mellitus inadequately controlled with diet and exercise. Diabetes, obesity & metabolism 15 (4):372-382. doi:10.1111/dom.12054

59. Strojek K, Yoon KH, Hruba V, Elze M, Langkilde AM, Parikh S (2011) Effect of dapagliflozin in patients with type 2 diabetes who have inadequate glycaemic control with glimepiride: a randomized, 24-week, double-blind, placebo-controlled trial. Diabetes, obesity & metabolism 13 (10):928-938. doi:10.1111/j.1463-1326.2011.01434.x

60. Strojek K, Yoon KH, Hruba V, Sugg J, Langkilde AM, Parikh S (2014) Dapagliflozin added to glimepiride in patients with type 2 diabetes mellitus sustains glycemic control and weight loss over 48 weeks: a randomized, double-blind, parallel-group, placebo-controlled trial. Diabetes therapy : research, treatment and education of diabetes and related disorders 5 (1):267-283. doi:10.1007/s13300-014-0072-0

61. Terra SG, Focht K, Davies M, Frias J, Derosa G, Darekar A, Golm G, Johnson J, Saur D, Lauring B, Dagogo-Jack S (2017) Phase III, efficacy and safety study of ertugliflozin monotherapy in people with type 2 diabetes mellitus inadequately controlled with diet and exercise alone. Diabetes, obesity & metabolism 19 (5):721-728. doi:10.1111/dom.12888

62. Wilding JP, Woo V, Rohwedder K, Sugg J, Parikh S, Dapagliflozin 006 Study G (2014) Dapagliflozin in patients with type 2 diabetes receiving high doses of insulin: efficacy and safety over 2 years. Diabetes, obesity & metabolism 16 (2):124-136. doi:10.1111/dom.12187

63. Wilding JP, Woo V, Soler NG, Pahor A, Sugg J, Rohwedder K, Parikh S, Dapagliflozin 006 Study G (2012) Long-term efficacy of dapagliflozin in patients with type 2 diabetes mellitus receiving high

doses of insulin: a randomized trial. Ann Intern Med 156 (6):405-415. doi:10.7326/0003-4819-156-6-201203200-00003

64. Wilding JP, Charpentier G, Hollander P, Gonzalez-Galvez G, Mathieu C, Vercruysse F, Usiskin K, Law G, Black S, Canovatchel W, Meininger G (2013) Efficacy and safety of canagliflozin in patients with type 2 diabetes mellitus inadequately controlled with metformin and sulphonylurea: a randomised trial. Int J Clin Pract 67 (12):1267-1282. doi:10.1111/ijcp.12322

65. Wiviott SD, Raz I, Bonaca MP, Mosenzon O, Kato ET, Cahn A, Silverman MG, Zelniker TA, Kuder JF, Murphy SA, Bhatt DL, Leiter LA, McGuire DK, Wilding JPH, Ruff CT, Gause-Nilsson IAM, Fredriksson M, Johansson PA, Langkilde AM, Sabatine MS, Investigators D-T (2019) Dapagliflozin and Cardiovascular Outcomes in Type 2 Diabetes. The New England journal of medicine 380 (4):347-357. doi:10.1056/NEJMoa1812389

66. Yale JF, Bakris G, Cariou B, Yue D, David-Neto E, Xi L, Figueroa K, Wajs E, Usiskin K, Meininger G (2013) Efficacy and safety of canagliflozin in subjects with type 2 diabetes and chronic kidney disease. Diabetes, obesity & metabolism 15 (5):463-473. doi:10.1111/dom.12090

67. Yang W, Han P, Min KW, Wang B, Mansfield T, T'Joen C, Iqbal N, Johnsson E, Ptaszynska A (2016) Efficacy and safety of dapagliflozin in Asian patients with type 2 diabetes after metformin failure: A randomized controlled trial. Journal of diabetes 8 (6):796-808. doi:10.1111/1753-0407.12357

68. Yang W, Ma J, Li Y, Zhou Z, Kim JH, Zhao J, Ptaszynska A (2018) Dapagliflozin as add-on therapy in Asian patients with type 2 diabetes inadequately controlled on insulin with or without oral antihyperglycemic drugs: a randomized controlled trial. Journal of diabetes (no pagination). doi:10.1111/1753-0407.12634

69. Zinman B, Wanner C, Lachin JM, Fitchett D, Bluhmki E, Hantel S, Mattheus M, Devins T, Johansen OE, Woerle HJ, Broedl UC, Inzucchi SE (2015) Empagliflozin, Cardiovascular Outcomes, and Mortality in Type 2 Diabetes. N Engl J Med 373 (22):2117-2128. doi:10.1056/NEJMoa1504720
